# Bacterial Porin Disrupts Mitochondrial Membrane Potential and Sensitizes Host Cells to Apoptosis

**DOI:** 10.1371/journal.ppat.1000629

**Published:** 2009-10-23

**Authors:** Vera Kozjak-Pavlovic, Elke A. Dian-Lothrop, Michael Meinecke, Oliver Kepp, Katharina Ross, Krishnaraj Rajalingam, Anke Harsman, Eva Hauf, Volker Brinkmann, Dirk Günther, Ines Herrmann, Robert Hurwitz, Joachim Rassow, Richard Wagner, Thomas Rudel

**Affiliations:** 1 Department of Molecular Biology, Max Planck Institute for Infection Biology, Berlin, Germany; 2 Biocenter, Chair of Microbiology, University of Würzburg, Würzburg, Germany; 3 Institute for Physiological Chemistry, Ruhr-University Bochum, Bochum, Germany; 4 Department of Biology/Chemistry, Division of Biophysics, University of Osnabrück, Osnabrück, Germany; 5 Protein Purification Core Facility, Max Planck Institute for Infection Biology, Berlin, Germany; 6 Microscopy Core Facility, Max Planck Institute for Infection Biology, Berlin, Germany; Northwestern University Feinberg School of Medicine, United States of America

## Abstract

The bacterial PorB porin, an ATP-binding β-barrel protein of pathogenic *Neisseria gonorrhoeae*, triggers host cell apoptosis by an unknown mechanism. PorB is targeted to and imported by host cell mitochondria, causing the breakdown of the mitochondrial membrane potential (ΔΨ_m_). Here, we show that PorB induces the condensation of the mitochondrial matrix and the loss of cristae structures, sensitizing cells to the induction of apoptosis via signaling pathways activated by BH3-only proteins. PorB is imported into mitochondria through the general translocase TOM but, unexpectedly, is not recognized by the SAM sorting machinery, usually required for the assembly of β-barrel proteins in the mitochondrial outer membrane. PorB integrates into the mitochondrial inner membrane, leading to the breakdown of ΔΨ_m_. The PorB channel is regulated by nucleotides and an isogenic PorB mutant defective in ATP-binding failed to induce ΔΨ_m_ loss and apoptosis, demonstrating that dissipation of ΔΨ_m_ is a requirement for cell death caused by neisserial infection.

## Author Summary

PorB is a bacterial porin that plays an important role in the pathogenicity of *Neisseria gonorrhoeae*. Upon infection with these bacteria, PorB is transported into mitochondria of infected cells, causing the loss of mitochondrial membrane potential and eventually leading to apoptotic cell death. Here, we show that PorB enters mitochondria through the TOM complex, similar to other mitochondria-targeted proteins, but then bypasses the SAM complex machinery that assembles all other porin-like proteins into the outer mitochondrial membrane. This leads to the accumulation of PorB in the intermembrane space and the integration of a fraction of PorB into the inner mitochondrial membrane (IMM). In the IMM, ATP-regulated pores are formed, leading to dissipation of membrane potential and the loss of cristae structure in affected mitochondria, the necessary first steps in induction of apoptosis. Our work offers, for the first time, a detailed analysis of the mechanism by which PorB targets and damages host cell mitochondria.

## Introduction

The genus *Neisseria* is comprised of the human pathogenic species *N. gonorrhoeae* (Ngo) and *N. meningitidis*, which cause gonorrhea and meningitis, respectively. The attachment of bacteria to epithelial cells results in transfer of the outer membrane porin PorB to the host cell cytoplasmic membrane [1],[2] and mitochondria [3],[4]. Infection by Ngo causes loss of membrane potential (ΔΨ_m_) across the inner mitochondrial membrane (IMM) and release of cytochrome *c*, which is required for activation of caspases and induction of apoptosis [5]. When expressed in host cells, PorB translocates to mitochondria and efficiently causes the breakdown of ΔΨ_m,_ but fails to induce the release of cytochrome *c* and subsequent apoptosis under these conditions [6]. This suggested that PorB is required, but is not sufficient, to induce apoptosis, and that a second signal is needed to induce cytochrome *c* release during infection.

Pro-apoptotic signals like growth factor withdrawal, DNA damage or cytoskeletal rearrangement lead to the activation of so-called BH3-only proteins, pro-apoptotic members of the Bcl-2 family [7]. Active BH3-only proteins cause the oligomerization and pore formation of Bax and Bak in the outer mitochondrial membrane (OMM) [8],[9]. We recently demonstrated that signaling cascades originating from the initial interaction of gonococci with host cells specifically induce the release of the cytoskeletal associated proteins Bim and Bmf, which are both required for the full induction of apoptosis by gonococcal infection [10]. Bim and Bmf activate proapoptotic Bak and Bax proteins, inducing OMM perforation followed by the release of caspase-activating factors into the cytosol and activation of apoptosis [11]. Thus, Bim- and Bmf-initiated events may act in cooperation with mitochondrial PorB in apoptosis induction.

Whereas targeting of PorB to mitochondria and its crucial role in *Ngo*-induced apoptosis are well established [3],[6], the molecular mechanism by which PorB causes loss of ΔΨ_m_ remains unknown. Studies with yeast mitochondria have indicated that import of PorB, and also other bacterial porins, into mitochondria might follow the same pathway as the endogenous mitochondrial porin, voltage-dependent anion-selective channel (VDAC) [6],[12]. In addition, as bacterial PorB and mitochondrial VDAC are both classical β-barrel proteins, structural similarities may facilitate recognition of bacterial PorB by the mitochondrial protein import machinery.

In general, uptake of newly synthesized proteins from the cytosol is mediated by the TOM complex, the translocase of the mitochondrial outer membrane [13]. Within this complex, the general import pore is formed by Tom40 [14]. At the OMM of yeast mitochondria, both endogenous VDAC [15] and a PorB derivative [6] target Tom40 to enter mitochondria. VDAC and all other mitochondrial β-barrel proteins tested so far are subsequently transferred to the SAM/TOB complex (sorting and assembly machinery) in the mitochondrial outer membrane [16],[17]. Interestingly, the core component of this complex Sam50/Tob55, shows remarkable similarities to the bacterial outer membrane protein Omp85 [18]; Omp85 has been shown to mediate membrane insertion of PorB and other β-barrel proteins in *Neisseria meningitidis* [19],[20].

Considering the obvious homologies between the Omp85 family members [18], bacterial PorB should be recognized and inserted into the OMM by the SAM/TOB complex. This would also be in agreement with the general rule that β-barrel proteins are found neither in bacterial nor in mitochondrial inner membranes. However, if PorB accumulates in the OMM, it is difficult to explain how it dissipates ΔΨ_m_, since this would require massive ion flux across the IMM.

Here, we investigated the role of mitochondrial targeting of PorB during the course of infection-induced apoptosis. Our data demonstrate that the cooperation of PorB and signaling pathways activated by BH3-only proteins induces the release of cytochrome *c* and the activation of caspases. We show that PorB avoids Sam50/Tob55 and Sam37/Mas37, two core components of the SAM/TOB complex, to integrate into the IMM. As a result, mitochondria lose their ΔΨ_m_ and the structural integrity of the cristae is dramatically altered. We propose that these modifications at the IMM are essential early events in Ngo-induced apoptosis.

## Results

### PorB cooperates with BH3-only proteins to induce cytochrome *c* release and caspase activation

**Figure 1:**
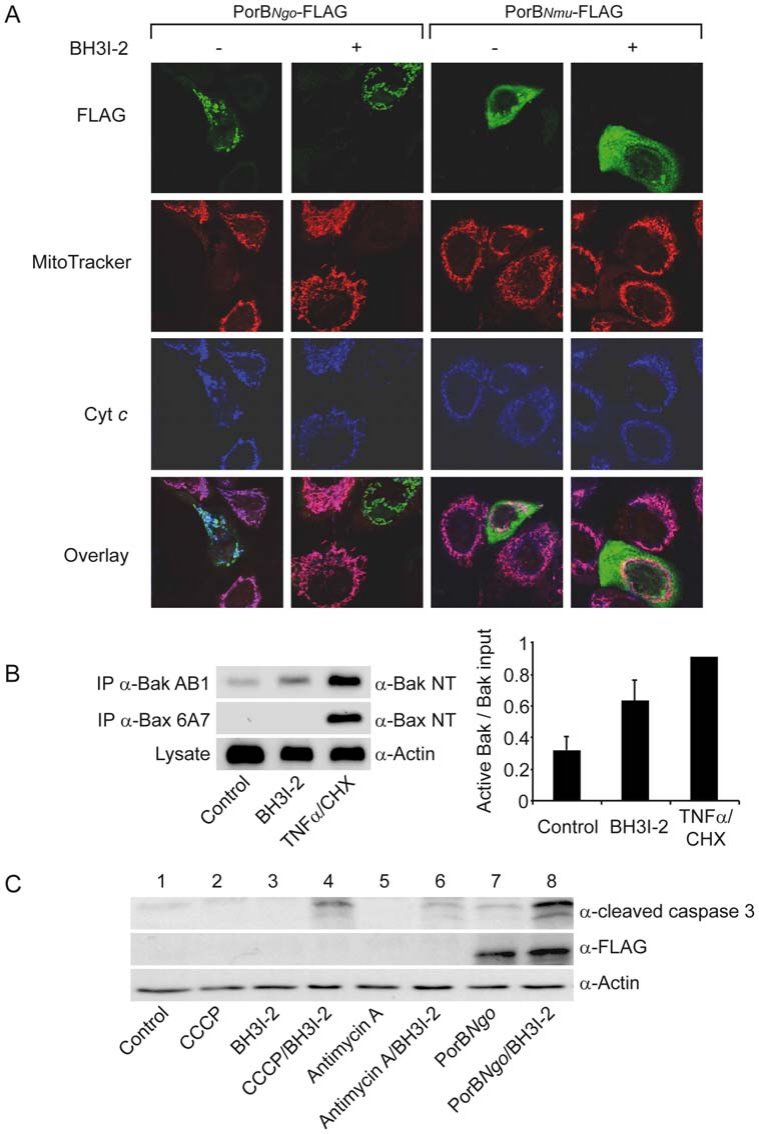
PorB and BH3-only protein-induced signaling pathways cooperate to activate caspases. (A) FLAG-tagged porins from *N. gonorrhoeae* (PorB*_Ngo_*-FLAG) and commensal *N. mucosa* (PorB*_Nmu_*-FLAG) were expressed in HeLa cells. Shown are the overlays of FLAG-tag (green), and cytochrome *c* (blue). The mitochondria with membrane potential were detected using MitoTracker staining (red). 25 µM BH3-mimetic compound BH3I-2 (Calbiochem) was added 24 h post transfection where indicated. (B) BH3I-2- or TNFα/cycloheximide (TNFa/CHX)-treated cells were subjected to immunoprecipitation with conformation specific antibodies recognizing activated Bak or Bax. Graph depicts quantities of precipitated Bak, Bax and actin (loading control), which were detected by western blot. Data are mean±SD of three independent experiments. (C) Caspase-3 activation was detected by western blot analysis upon treatment with BH3I-2 as in A. Cells were treated with either protonophore CCCP (Sigma) or the complex III inhibitor antimycin A (Sigma) at 1 µM as positive controls. Blot is representative of three independent experiments.

Our previous observations that PorB of pathogenic *Neisseria* efficiently targeted mitochondria and induced ΔΨ_m_ loss without triggering the release of cytochrome *c* ([3] and Fig. 1A), confirmed that these were independent processes, at least in our model. Therefore, we reasoned that PorB-expressing HeLa cells lack signals upstream of mitochondria to efficiently release cytochrome *c*. Such potential upstream signals are mediated by BH3-only proteins, which we recently identified as necessary factors for gonococci-induced apoptosis [10]. To demonstrate the interplay of PorB-triggered ΔΨ_m_ loss and BH3-only protein-induced pathways for the release of proapoptotic factors, we activated Bak in HeLa cells using the cell permeable BH3-mimetic compound BH3I-2. BH3I-2 activates Bak (Fig. 1B) by interfering with its binding to Bcl-X_L_ [21]. We then transfected HeLa cells with the *porB* gene of strain VPI (PorB*_Ngo_*) and monitored PorB expression, ΔΨ_m_ and cytochrome *c* distribution, using immunofluoresence microscopy. As a control, PorB*_Nmu_* of the commensal strain *N. mucosa*, which does not target mitochondria [6], was expressed. The mitochondria of cells expressing PorB*_Nmu_* were unchanged in comparison to control cells; in contrast, those expressing PorB*_Ngo_* lost ΔΨ_m_ but stained positive for cytochrome *c* (Fig. 1A), as previously described [6]. Addition of BH3I-2 had no effect on either the membrane potential or the cytochrome *c* content of mitochondria of PorB*_Nmu_*-expressing cells (Fig. 1A). Only PorB*_Ngo_*-expressing cells stained negative for cytochrome *c* in the presence of BH3I-2 (Fig. 1A), suggesting that mitochondrial targeting of PorB*_Ngo_* sensitizes cells for the complete release of cytochrome *c* upon Bak activation.

To test whether BH3I-2 treatment induces caspase cleavage and activation in PorB*_Ngo_*-expressing cells, western blot analysis was performed to detect active caspase 3. Caspase 3 remained inactivated in cells treated only with BH3I-2 (Fig. 1C, lane 3). PorB*_Ngo_* expression alone resulted in minimal caspase activity; however, upon addition of BH3I-2 a sharp increase in activity was elicited (Fig. 1C, lanes 7 and 8). Interestingly, dissipation of ΔΨ_m_ by treatment of cells with the uncoupling reagent CCCP (carbonyl cyanide *m*-chlorophenyl hydrazone) or Antimycin A, an inhibitor of the electron transport chain, also caused activation of caspases when combined with BH3-I2 treatment (Fig. 1C, lanes 4 and 6), although to a lesser extent. These results suggested that PorB*_Ngo_*-induced ΔΨ_m_ loss facilitates the release of cytochrome *c*, which in turn activates caspase-3.

### PorB induces mitochondrial matrix condensation and loss of cristae structure

**Figure 2:**
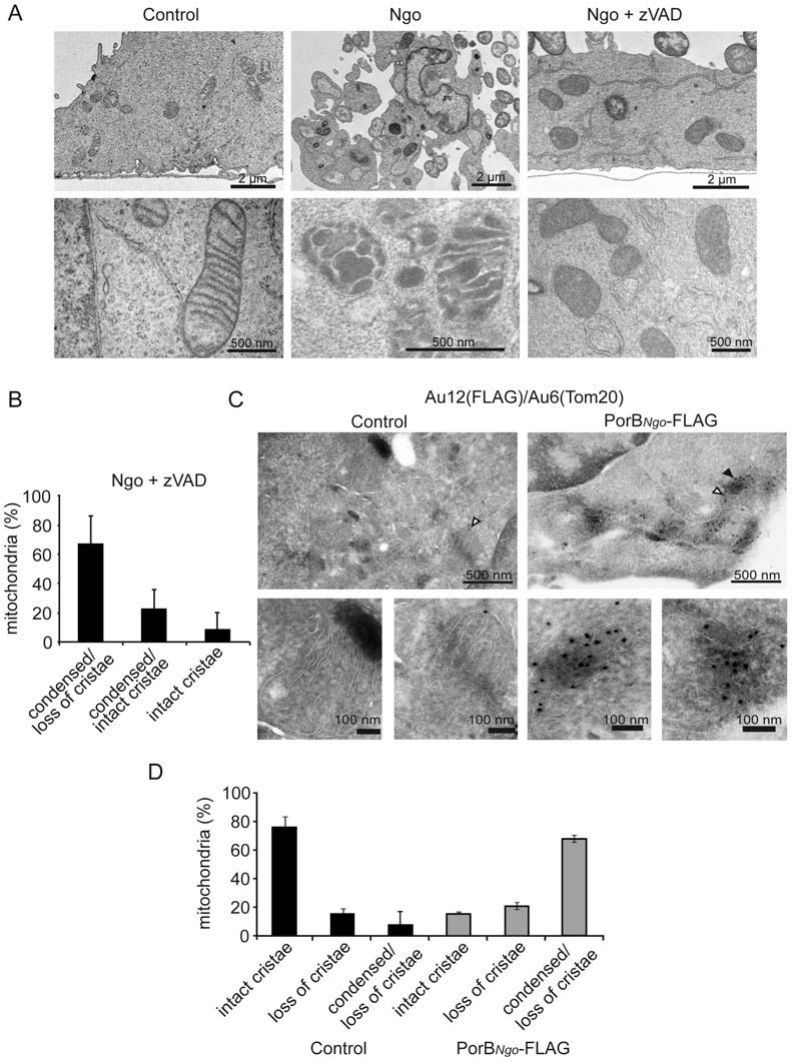
PorB-induced rearrangement of the inner mitochondrial membrane. (A) HeLa cells infected in the presence or absence of zVAD were analyzed by EM to detect the structural changes in the mitochondria. Representative sections of control, infected (Ngo) and infected, zVAD treated cells (Ngo+zVAD) are shown (upper panel). The mitochondria were viewed at higher magnification to analyze alterations in the cristae architecture (lower panel). (B) A total of 500 mitochondria were counted from cells infected and pretreated with zVAD and analyzed for condensation and loss of cristae. The graph shows the relative number of mitochondria with the indicated phenotypes of all analyzed mitochondria. Data are mean±SD of three independent experiments. (C) Transfection of *porB* leads to loss of cristae and matrix condensation. FLAG-tagged PorB was detected by immunogold labeling with a primary FLAG antibody and a secondary antibody linked to 12 nm gold particles (filled arrow head). Endogenous protein of the OMM (Tom20) was co-stained with the respective antisera and a secondary antibody linked to 6 nm gold particles (open arrow head). (D) Normal mitochondria or mitochondria with condensed matrix and disrupted cristae were quantified in PorB-expressing and control cells and expressed as % of total mitochondria. Data are mean±SD of three different experiments.

The release of cytochrome *c* during apoptosis is a multi-step process that requires a complete remodeling of the IMM [22]. Loss of cristae structure and subsequent condensation of the mitochondrial matrix is often detected in mitochondria devoid of ΔΨ_m_ [23],[24]. Accordingly, we tested whether infection and/or PorB expression induce remodeling of mitochondria. Electron microscopy (EM) revealed that most of the mitochondria in infected apoptotic cells were highly condensed and appeared dark (Fig. 2A), a phenomenon explained by an increased electron diffraction of the condensed matrix. When HeLa cells were treated with the caspase inhibitor zVAD prior to infection, at least 60% of the mitochondria from these cells displayed a dark matrix and loss of cristae (Fig. 2A,B). Immunogold-labeling of PorB-transfected cells revealed that most PorB containing mitochondria underwent extensive condensation of the matrix and loss of cristae structure (Fig. 2C,D). We therefore conclude that the presence of PorB triggers a major reorganization of the IMM.

### Uptake of PorB is mediated by the mitochondrial TOM complex, but is independent of the SAM

Since induction of ΔΨ_m_ loss is most likely the crucial step in the sensitization of mitochondria by PorB and gonococcal infection, we investigated the underlying mechanisms of this process. Since PorB is structurally similar to other β-barrel proteins, it should be recognized by the SAM/TOB complex [25],[26] and insert into the OMM. To test this assumption, we isolated mitochondria from HeLa cells, incubated them with radiolabelled PorB and subsequently subjected these mitochondria to carbonate extraction at different stringencies (pH 10.8 and 11.5) to remove loosely attached PorB. The membrane protein VDAC was present in the pellet at both pH, but the soluble protein Hsp60 was found completely in the supernatant only at more stringent conditions, at pH 11.5. Although the amount of soluble PorB increased with the increase of stringency of carbonate extraction, a fraction of radiolabelled PorB remained associated with membranes even after carbonate extraction at pH 11.5 (Fig. S1A). Upon import into mitochondria, the amount of carbonate-resistant PorB increased with time, but only traces of PorB were detected in the pellet after carbonate-extraction of mock samples containing no mitochondria (Fig. S1B). Thus, carbonate-resistant PorB is not formed independently of mitochondria, but instead is a fully imported and membrane-integrated fraction.

**Figure 3:**
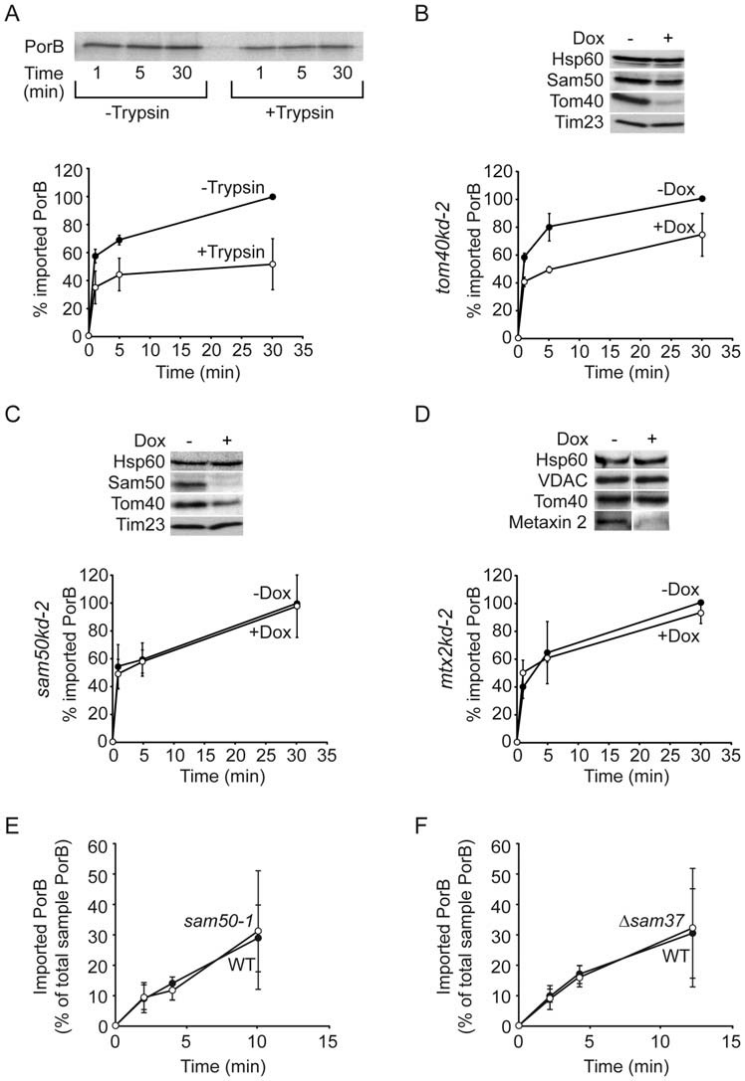
Import of PorB into isolated mitochondria. (A) Mitochondria were pretreated with 20 µg/ml of trypsin in the presence (−Trypsin) or absence (+Trypsin) of 600 µg/ml Soybean trypsin inhibitor (SBTI). In the latter (+Trypsin) trypsin was subsequently inactivated by addition of 600 µg/ml SBTI. Treated mitochondria were incubated with a radiolabelled PorB precursor for indicated times followed by carbonate extraction at pH 11.5. The graph depicts mean±SD of three independent import experiments. Maximum PorB import time into non- Trypsin treated mitochondria was set at 100%. (B,C,D) Mitochondria were isolated from *tom40kd-2*, *sam50kd-2* and *mtx2kd-2* cell lines grown in the absence (−Dox) or the presence (+Dox) of doxycycline for 7 days, except for *tom40kd-2* cells which were treated with Dox for 5 days. After the import of radiolabelled PorB for indicated times, mitochondria were subjected to carbonate extraction at pH 11.5 and SDS-PAGE. Graph depicts mean±SD of three independent experiments. Maximum PorB import time into mitochondria in cells not treated with Dox was set at 100%. Immunoblots demonstrate the levels of import factors in the mitochondria used for import assays and are representative of three independent experiments. (E,F) Import of ^35^S-labelled *N. gonorrhoeae* PorB into mitochondria isolated from the yeast mutant strain *sam50-1*, *Δsam37* strain and from the corresponding wildtype strains. The amount of imported PorB was normalized against the total amount of PorB in the import reaction. Graph depicts mean±SD of four independent experiments.

To determine if OMM proteins are involved in the uptake of PorB, we pretreated the isolated mitochondria with trypsin to remove cytosolic domains of OMM proteins and compared the rate of import in pretreated and untreated mitochondria. The amount of carbonate-resistant PorB after import was significantly reduced in trypsin-pretrated mitochondria, reaffirming the dependence of PorB import on the TOM complex (Fig. 3A, Fig. S1E and [6]). Likewise, PorB import was reduced (by 20–30%) in mitochondria of *tom40kd-2* cells after 5 days of knockdown induction with Dox (Fig. 3B). Of note, even after knockdown, traces of Tom40 that can function in import are still present in the mitochondria (Fig. 3B and not shown). Importantly, PorB import into mitochondria isolated from *pLVTHM* cells containing an empty vector was unchanged upon treatment with Dox, excluding a Dox-dependent side effect (not shown). In addition, we have previously shown that import of VDAC into mitochondria isolated from *tom40kd-2* cells was reduced, along with other TOM components, including Tom20 and Tom22 [27]. Hence, our findings support the notion that the TOM complex is involved in the import of both VDAC and PorB.

We then addressed the possible role of the SAM complex in the import of PorB using HeLa-derived cell lines with inducible knockdown of Sam50 (*sam50kd-2*) and Metaxin 2 (*mtx2kd-2*) [27], a putative mammalian homologue of yeast Sam35. VDAC import into the mitochondria isolated from these cell lines was clearly restricted after the induction of knockdown, confirming our previous data (Fig. S2A,B), [27]) and demonstrating the functional downregulation of Sam50 and Metaxin 2 in these mitochondria. Contrary to our expectations, in Sam50- or Metaxin 2-depleted mitochondria, PorB import was unimpeded (Fig. 3C,D). As observed before [27], in mitochondria with the knockdown of Sam50, levels of Tom40 were likewise reduced in the range of 40–50% (Fig. 3C). However, we did not see any decrease of PorB import into these mitochondria in spite of the reduction of Tom40 amounts. This can be explained by the fact that even a strong Tom40 knockdown of more than 90% affected PorB import only moderately (Fig. 3B); a Tom40 reduction of 40–50% might simply not be sufficient to cause any effects.

Previously published data had suggested that PorB follows the same import route as VDAC [6]. However, our data indicated that the SAM/TOB complex marks a crucial branching point of the pathways. To investigate further, we used a similar approach to most previous studies [25],[26] and used yeast to test whether PorB is a substrate of the SAM complex. PorB transport was monitored in isolated yeast mitochondria using radiolabelled PorB. The protease-protected fraction of PorB was only obtained in the presence of mitochondria (Fig. S1C), ruling out a non-specific aggregation of PorB and confirming import into the mitochondria. As reported previously, the homologous PorB of the non-pathogenic strain *N. mucosa* was not imported into mitochondria (Fig. S1D; [6]). PorB and other β-barrel protein import efficiencies into mitochondria were then monitored. Similar to our findings with human mitochondria (Fig. 3B,C,D), PorB import efficiencies were comparable to WT levels in the bacterial mutant *sam50-1* strain (Fig. 3E), whereas in the yeast mutant *tom40-4* import was reduced to levels reported for the mitochondrial porin VDAC [15] (Fig. S1E). Import of VDAC into *sam50-1* mitochondria was impaired (Fig. S2C); in contrast, import of the IMM dicarboxylate carrier (DIC) remained unchanged (Fig. S2D), supporting a specific role for the SAM complex in the transport of OMM β-barrel proteins [17],[28]. Similar results were obtained with mitochondria isolated from a *Δsam37* strain lacking the Metaxin 1 homologue Sam37/Mas37 [16],[29] (Fig. 3G, S2E & S2F), an essential factor for the mitochondrial import of β-barrel proteins [16]. Collectively, these findings suggest that PorB, although a β-barrel protein, avoids the SAM complex during import into mitochondria.

### Transport of PorB to the inner mitochondrial membrane

**Figure 4:**
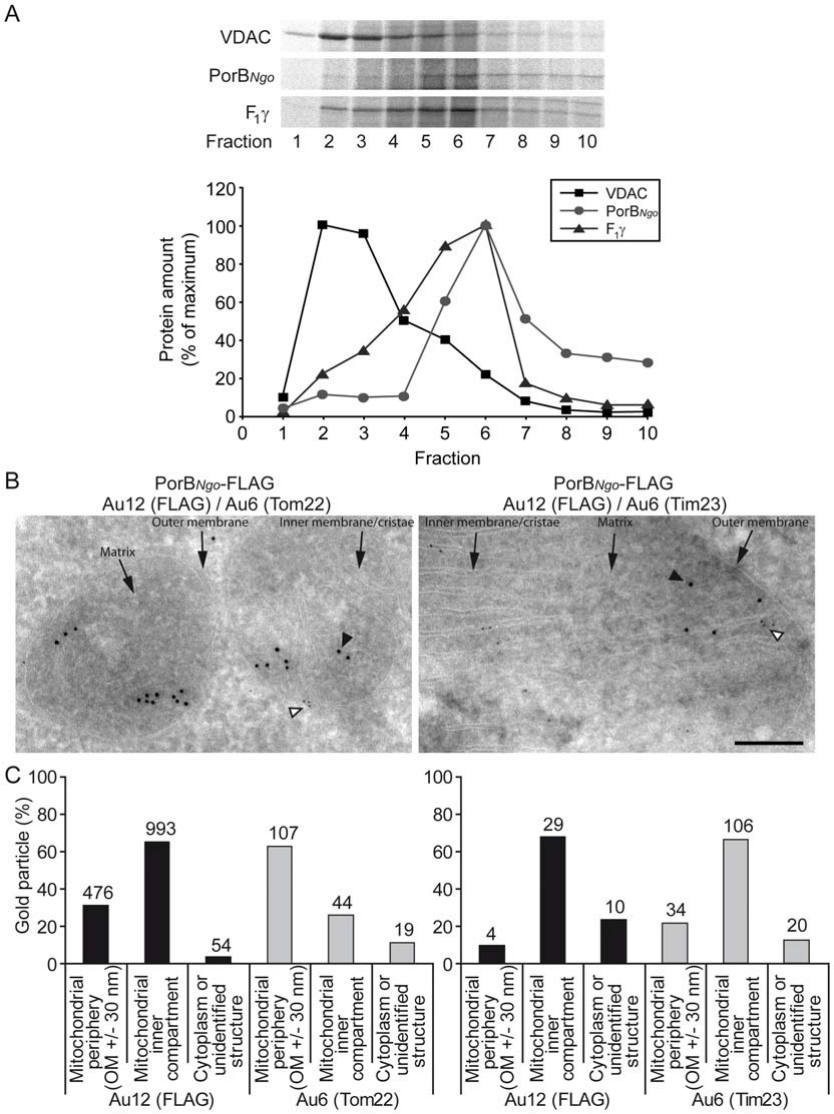
Association of PorB with the mitochondrial inner membrane. (A) Sucrose density gradient. ^35^S-labelled PorB, together with the marker proteins VDAC (outer membrane) and F1γ (the ATP-synthase γ-subunit, inner membrane), were synthesized in reticulocyte lysate and imported into isolated yeast mitochondria. Following treatment with proteinase K, membrane vesicles were prepared by sonication and separated on a linear sucrose gradient (0.85–1.6 M sucrose). The fractions were analyzed by SDS-PAGE and a phosphorimager. The highest value for each protein was set at 100% (control). (B) Localization of PorB at the IMM in HeLa cells. HeLa cells were transfected with an expression construct of FLAG-tagged PorB. PorB was detected in *porB*-transfected cells by immunogold labelling using an antibody directed against the FLAG-tag and a secondary antibody linked to 12 nm gold particles. Endogenous proteins of the OMM (Tom22) and IMM (Tim23) were co-stained with the respective antisera and a secondary antibody linked to 6 nm gold particles. Arrows represent different features of mitochondria (outer membrane, inner membrane and matrix). Arrowheads show 12 nm (closed arrowheads) or 6 nm (open arrowheads) gold particles on electronmicrographies. (C) Quantification of (B). Gold particles were counted to determine the intramitochondrial localization of PorB, Tom22 and Tim23. The actual number of counted gold particles is indicated above the individual bars.

To determine the precise intramitochondrial location of PorB, we prepared highly purified outer membrane vesicles from yeast mitochondria [30]. Unexpectedly, PorB was not present in the vesicles but only in the pellet fraction (Fig. S3, lane OMV vs. lane MPF). In fact, using these conditions PorB mainly formed aggregates in the cytosol, compromising further analysis. Therefore, we first imported radiolabelled PorB into mitochondria isolated from the WT strain, and then separated mitochondrial membrane vesicles by sucrose density centrifugation. Imported PorB accumulated partially in a carbonate-resistant form, as was the case in human mitochondria, indicating its integration into the membrane (Fig. S1A and data not shown). Whereas PorB accumulated in higher density fractions of the gradient, the other β-barrel protein, OMM porin VDAC, was found in the upper part of the gradient. The majority of PorB accumulated in the same higher density fraction as the γ-subunit of the IMM ATP synthase (F_1_γ) (Fig. 4A). This fractionation pattern clearly demonstrated that PorB does not accumulate in the OMM and that PorB is at least partially associated with the IMM; however, a significant fraction of the newly imported PorB seems to form aggregates, as suggested by the presence of PorB in the high density fractions 8, 9, and 10 (Fig. 4A).

Cytosolic PorB aggregates have never been observed in mammalian cells, suggesting that they express PorB in a fully import-competent state *in vivo*. The immediate dissipation of ΔΨ_m_ in HeLa cells shows that PorB enters the mitochondria in an active form [6]. We expressed PorB in HeLa cells and determined its sub-mitochondrial localization by immunogold electron microscopy. The vast majority of the PorB gold particles localized to the IMM and matrix, similar to the endogenous IMM protein Tim23 (Fig. 4B,C). For comparison with an endogenous OMM marker, we included labeling of Tom22, which showed a clearly different distribution (Fig. 4B,C). Taken together, these findings show that PorB avoids interactions with the SAM machinery and is instead directed to the intermembrane space (IMS). The distribution of PorB in HeLa cells indicates that, at least in human mitochondria, PorB preferentially associates with the IMM.

### PorB forms high conductance pores

**Figure 5:**
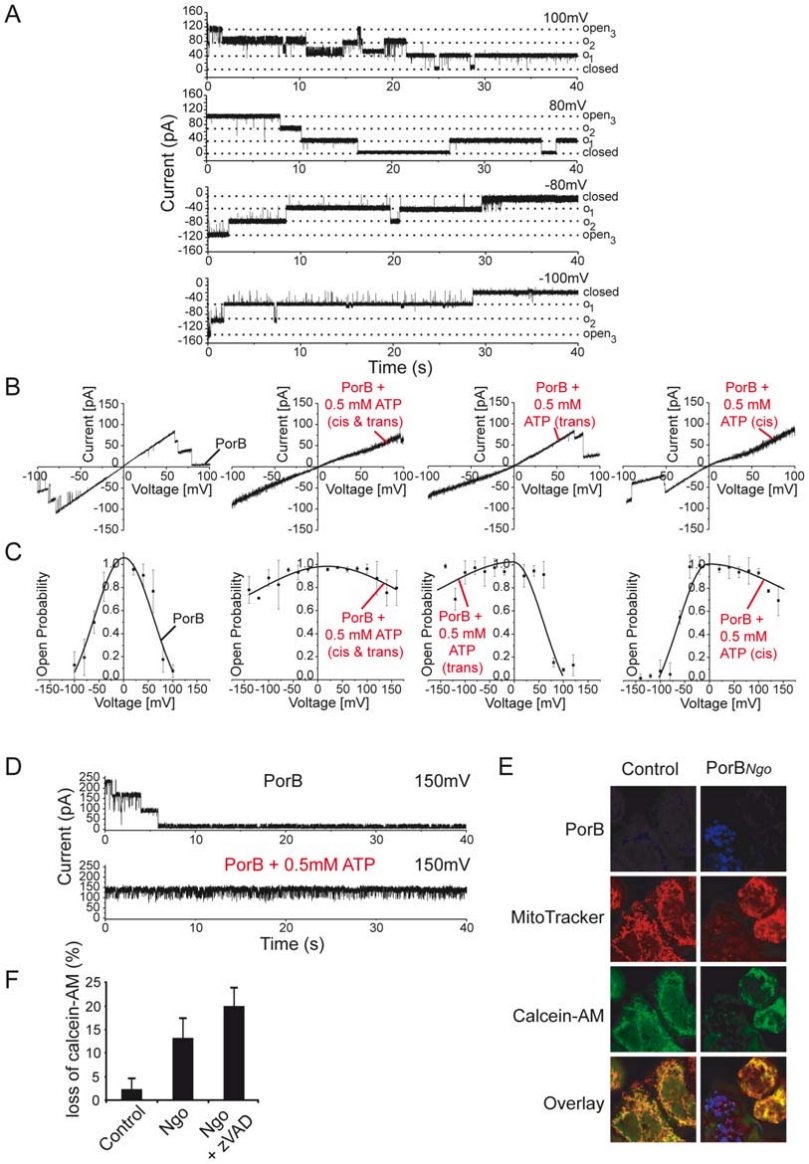
Electrophysiological characterization of PorB. (A) Typical (n≥5 independent bilayers) current recordings of a bilayer containing PorB at indicated applied voltages. Buffer conditions were symmetrical with 250 mM KCl, 10 mM Mops/Tris pH 7.0 in *cis* and *trans*. (B) PorB is regulated by ATP. Current-voltage relationship of PorB in the absence or presence of ATP added at the indicated side of the membrane. Buffer conditions were as in (A) (n≥3). (C) Voltage-dependent open probability of PorB in the absence or presence of ATP added to the indicated side of the membrane. Quantification was performed by comparing the mean current determined over a time range of 2 min with the maximum current at a constant holding potential (n≥3). (D) Current recordings of a bilayer containing PorB in the presence of ATP at the indicated constant holding potential. Buffer conditions were as in (A) (n≥5). (E) HeLa cells transfected with empty vector and *porB* were loaded with calcein-AM (green) and CoCl_2_ as mentioned in Protocol S1. The cells were then incubated with MitoTracker orange (red) to check for membrane potential loss, and the expression of PorB (blue) was checked by costaining. The respective overlays of all the three channels are shown. (F) The loss of calcein-AM staining upon Ngo infection was monitored by FACS analysis. Data represent mean±SD of three independent experiments.

It is known from previous multichannel studies that PorB is able to form pores of high conductance [2]. Whereas single channel data have been reported on *N. meningitidis* PorB [31], the investigations on gonococcal PorBs were restricted to multichannel recordings. However, only single channel analysis can clarify whether gonococcal PorBs can form a high-conductance channel in the IMM. Upon addition of purified PorB to either side of a planar lipid bilayer with lipid composition corresponding to the IMM [32], single-channel currents were readily detected (Fig. 5A). Although PorB exhibited a dynamic gating behavior with a multitude of conductance states (Fig. 5A & Fig. S4, insert), a main conductance state of Λ = 420±15 pS was also identified from a linear current-voltage relationship under symmetrical buffer conditions (Fig. S4). Previous observations with multi-channel recordings [2] indicated a voltage-dependent gating behavior of PorB, raising the question whether PorB channels remain open or closed under the physiological conditions present in the IMM (i.e. at ΔΨ_m_ of around 150 mV [33]). Gating transitions were rarely observed at lower voltages (not shown); PorB channels exhibited a typical three step channel closing at voltages above 60 mV (Fig. 5A). Considering the ΔΨ_m_ of the IMM and the voltage dependence of PorB, it is obvious that PorB channels incorporated into the IMM would in fact be arrested in a closed state and the capability of PorB to uncouple the mitochondria would be limited.

### Nucleotides regulate PorB channel currents

Since it is known that PorB shows an affinity for nucleotides [2] we analyzed the effect of ATP on PorB channels in detail. The concentration of ATP in mitochondria is known to range from 0.6 to 6.0 mM [34]. After addition of physiologically relevant amounts of ATP to PorB-containing bilayers, two intriguing effects were observed. First, the amplitude of the single-channel conductance was significantly reduced (Fig. 5A,B,D) accompanied by a drastic change in PorB gating behavior (Fig. 5A,D): The channel did not display the typical three step gating transitions but remained mainly in an open state exhibiting increased gating frequency, manifested as flickering (Fig. 5D). The second and physiologically even more important effect was the predominant loss of the voltage-dependent closure of PorB upon ATP addition (Fig. 5C). In intact mitochondria, where ATP is abundant, the channels would be forced to stay open even at a ΔΨ_m_ of 150 mV, allowing the flux of large currents across the IMM and subsequent rapid dissipation of ΔΨ_m_. The effects were verified by adding ATP to both sides of the membrane (Fig. 5B,C, left-most, centre-left) or separately to either the *trans* or the *cis* compartment, to test for side specificity (Fig. 5B,C, centre-right and rightmost). Since the usual channel closure is inhibited at the side of ATP addition and we do not know the exact orientation in which PorB incorporates into the membrane, it was important to establish that the effect of ATP on the PorB channel was not side-specific (Fig. 5B,C). These observations on single PorB channels allowed us to draw several important conclusions: (i) PorB inserts easily into a lipid bilayer, regardless of membrane potential. (ii) High concentrations of mitochondrial ATP stabilize the PorB channels in an open state. (iii) Considering a typical size and a simplified volume to surface ratio, a single open PorB channel should dissipate ΔΨ_m_ in about 0.8 ms (for details see Protocol S1).

### Permeabilization of the IMM upon PorB translocation

Next, we tested if PorB can perforate the IMM in living cells using a calcein quenching assay for infected and *porB* transfected cells (for details see Protocol S1). Transfection of PorB (Fig. 5E) or infection with Ngo (Fig. 5F & S5) led to the loss of calcein staining after cobalt chloride quenching, confirming IMM permeabilization upon PorB translocation. IMM permeabilization was not a consequence of apoptosis induction since preincubation of the cells with the caspase inhibitor zVAD-fmk failed to prevent IMM permeabilization or the loss of ΔΨ_m_ upon Ngo infection (Fig. 5F & S5). In conclusion, these data confirm that PorB is able to form pores in the IMM, leading to its permeabilization and loss of ΔΨ_m_.

### ATP binding by PorB is essential for the induction of ΔΨ_m_ loss and apoptosis by infection

**Figure 6:**
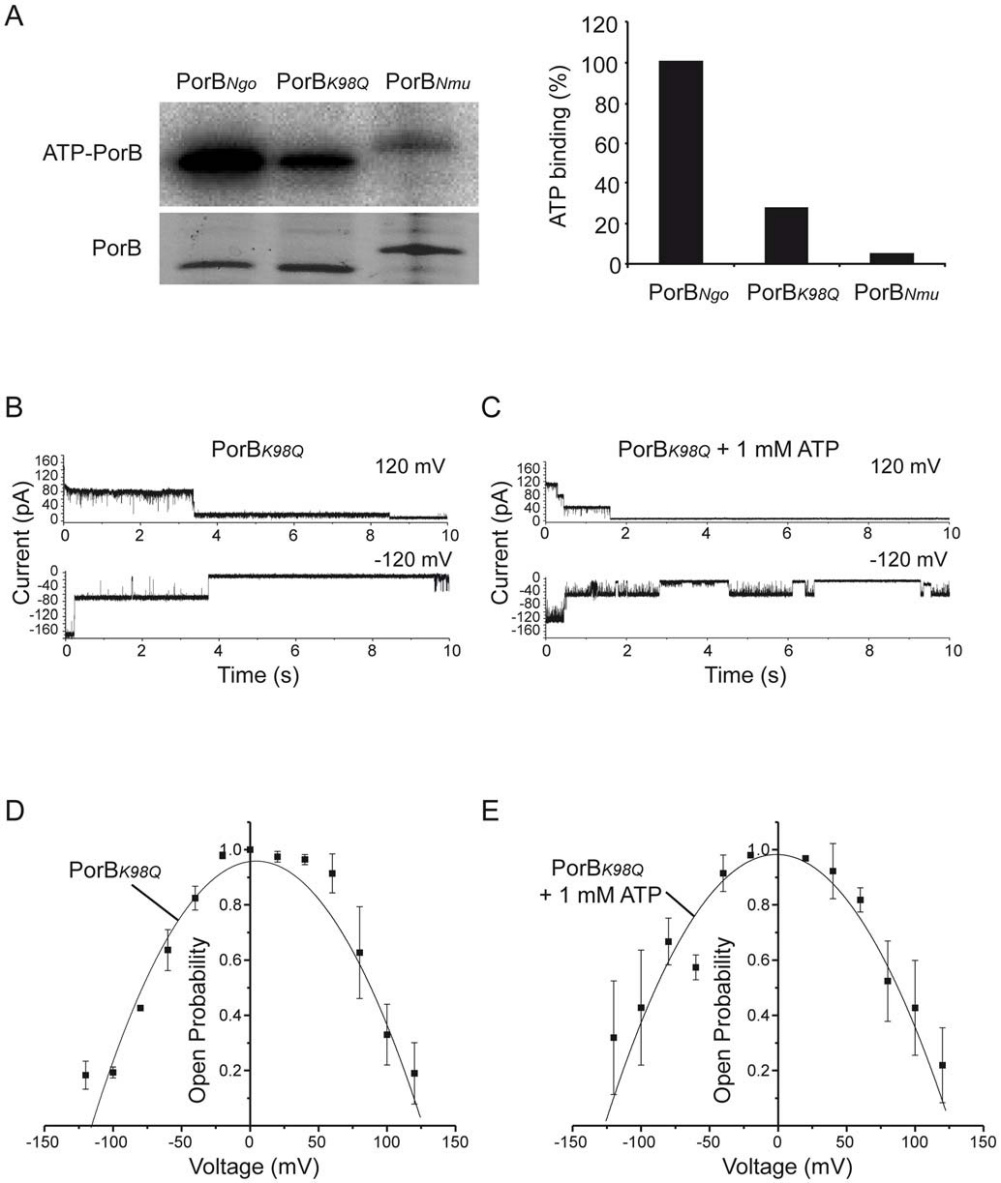
PorB*_K98Q_* channels are not modulated by ATP. (A) PorB*_K98Q_* is impaired in ATP binding. Neisserial strain N920 (PorB*_Ngo_*), N886 (PorB*_K98Q_*) and N923 (PorB*_Nmu_*) were subjected to ATP binding assay as described in Materials and Methods. Bacterial lysates were separated by SDS-PAGE, and labelled PorB was visualized by exposing the dried gel to X-Ray films. The lower panel represents PorB as visualized by coomassie staining. For quantification purposes, the amount of ATP bound by the strain N920 was taken to be 100%. (B,C) Typical (n≥5 independent bilayers) current recordings of a bilayer containing PorB*_K98Q_* at 120 mV and −120 mV. Buffer conditions were symmetric with 250 mM KCl, 10 mM Mops/Tris pH 7.0 in *cis* and *trans*. 1 mM ATP was added in *cis* and *trans* (C). (D,E) Voltage-dependent open probability of PorB*_K98Q_* in the absence (D) and presence (E) of ATP added to both sides of the membrane (n≥3). Data are mean±SD.

We have previously shown that PorB binds ATP via lysine residues potentially located in the PorB channel or the loop 3 region [2]. To identify the specific residues involved, we performed site-directed mutagenesis of several lysine residues to generate PorB derivatives deficient in ATP-binding. Mutant neisserial strains, differing only in the PorB derivative expressed [35], were tested for ATP binding as described previously [2]. Even though the exchange of lysine 98 for glutamine (PorB*_K98Q_*) reduced ATP-binding by more than 70% in comparison to WT PorB (PorB*_Ngo_*) (Fig. 6A), PorB*_K98Q_* still clearly localized to mitochondria in transfected cells (Fig. S6). Interestingly, ATP did not affect the channel properties of PorB*_K98Q_*. Neither the voltage-dependent gating of the PorB*_K98Q_* channel (Fig. 6B,C) nor the voltage-dependent open probability (Fig. 6D,E) was influenced by ATP, confirming that the effects of ATP on the PorB channel depend on lysine residue 98.

**Figure 7:**
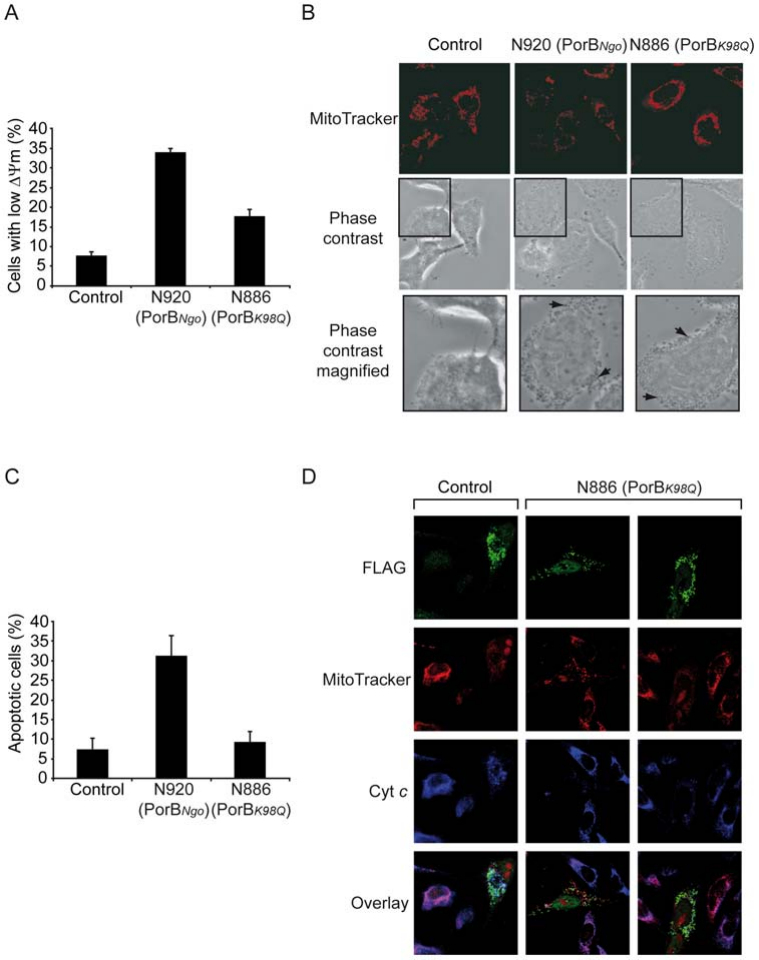
ATP binding is crucial for the PorB-induced ΔΨ_m_ loss and apoptosis. (A) PorB*_K98Q_* is impaired in inducing ΔΨ_m_ loss. HeLa cells were infected with the indicated strains N920 (containing wild type PorB*_Ngo_*) and N886 (containing ATP-binding mutant PorB*_K98Q_*), and the loss of ΔΨ_m_ was measured using TMRE staining and FACS analysis. The data are expressed as the relative number of cells with low TMRE staining from total cells. Data are mean±SD of 3 independent experiments. (B) HeLa cells were infected with the indicated strains and ΔΨ_m_ was monitored by MitoTracker staining. Phase contrast images demonstrate the strong infection of the HeLa cells. Arrows in the section of magnified phase contrast images point to bacteria. (C) HeLa cells were infected with the indicated strains, fixed and stained with 1 µg/ml Hoechst 33342 (Invitrogen) for 10 min followed by intense washing with PBS. A minimum of 200 cells from 5 randomly chosen fields per slide was analyzed for chromatin condensation using a Zeiss immunofluorescence microscope. Data are mean±SD of at least three independent experiments. (D) FLAG-tagged porin from *N. gonorrhoeae* (PorB*_Ngo_*-FLAG) was expressed in HeLa cells for 24 h prior to infection with strain N886. PorB*_Ngo_*-FLAG expression (green), cytochrome *c* (blue) and MitoTracker staining (red) were monitored. Note that infection with N886 triggered the release of cytochrome *c* only in cells with PorB*_Ngo_*-FLAG expression. Images are representative of three independent experiments.

To determine the effect of the K98Q mutation *in vivo*, we infected HeLa cells with isogenic neisserial strains harboring WT PorB*_Ngo_* (N920) and mutant PorB*_K98Q_* (N886) and then measured the loss of ΔΨ_m_ by using tetramethylrhodamine ethyl ester perchlorate (TMRE) staining and FACS analysis. Despite both strains having similar infection rates, significantly more cells infected with strain N886 carrying mutant PorB*_K98Q_* retained their ΔΨ_m_ as compared to cells infected with WT neisserial strain (Fig. 7A,B). Consistently, cells transfected with the PorB*_K98Q_* expression construct retained their ΔΨ_m_ in a number of cases (Fig. S6). Maintenance of ΔΨ_m_ was never observed with the WT PorB construct. Moreover, the potential of PorB*_K98Q_* mutant strain N886 to induce apoptosis in HeLa cells was strongly reduced (Fig. 7C) and infected cells failed to release cytochrome *c* from their mitochondria (Fig. 7D). When cells were transfected with WT PorB*_Ngo_* prior to infection with N886, they released cytochrome *c* (Fig. 7D), confirming that induction of ΔΨ_m_ loss and a second unknown signal triggered by *N. gonorrhoeae* infection are required for the induction of host cell apoptosis.

## Discussion

In this study, we demonstrate that PorB induces the reorganization of mitochondrial cristae and sensitizes mitochondria to release cytochrome *c* in response to infection and BH3-only protein induced signaling pathways. The initial prerequisite for PorB to sensitize infected cells to apoptosis is its ability to cause ΔΨm loss, probably by bypassing the OMM-SAM complex and accumulating in the intermembrane space.

Our previous data suggested that release of pro-apoptogenic factors by mitochondria during apoptosis induced by gonococcal infection requires two independent steps: The sensitization of mitochondria and the perforation of the OMM. The cooperative effect of PorB at the mitochondria and separate signals elicited by infected cells could be demonstrated in PorB-expressing cells during infection with strain N886, which carries the ATP-binding mutant of PorB, PorB*_K98Q_* (Fig. 7D). Our previous data also suggested that the infection-induced perforation of the OMM depends on the BH3-only proteins Bim and Bmf and the pro-apoptotic BH1-3 proteins Bak and Bax, activated by the interaction of the pathogen with host cells [10],[11]. The data presented here now demonstrate that mitochondria-targeted PorB in combination with a BH3-only mimetic compound is sufficient to induce cytochrome *c* release and the activation of caspases. This statement is supported by our observation that (i) only PorB*_Ngo_* targets the mitochondria and not the targeting-deficient derivative PorB*_Nmu_*, and (ii) only PorB*_Ngo_* recombinant cells and not the non-recombinant neighboring cells responded to BH3I-2 treatment. It is interesting to note that PorB from meningococcal strain H44/76 overexpressed in HeLa cells fails to induce ΔΨ_m_ dissipation but instead protects against apoptosis [36], whereas PorB from strain Z2491 has a similar uncoupling activity as gonococcal PorB [6]. Recent data suggest that invasive and carrier strains of *N. meningitides* from the same clonal complex induce or inhibit apoptosis, respectively. PorB from the invasive isolate promotes apoptosis induction [37], supporting a role for PorB in the life and death decision of *Neisseria*-infected cells.

As shown before [6], the amounts of PorB are relatively similar in mitochondria isolated from *Neisseria*-infected cells and cells transfected with PorB. However, the efficiency of transfection is approximately 30–40%, whereas the efficiency of infection is nearly 100%. Therefore, during transfection we estimate that 2- to 3-fold more PorB is present in a cell than during infection. Nevertheless, we observe the similar phenotype, a major reconstruction of cristae structures, in both infected and PorB recombinant cells. In this aspect, the transfection and infection model seem to be comparable.

The reconstitution of cristae structures is required for sensitization of mitochondria for the release of cytochrome c. Previous work has shown that the majority of cytochrome *c* is sequestered in the closed cristal compartments (85%); as a result structural changes in mitochondria are required to achieve complete and rapid release of cytochrome *c* [22]. Moreover, ΔΨ_m_ loss and matrix condensation contribute to cytochrome *c* release [24].

In the context of the biogenesis of endogenous mitochondrial proteins, it is not surprising that we find that the uptake of PorB into mitochondria is mediated by the TOM complex. This complex is involved in the import of practically all mitochondrial preproteins from the cytosol [38]–[40]. However, our observation that the import of PorB is independent from the SAM/TOB complex was unexpected. All previously tested endogenous mitochondrial β-barrel proteins, including VDAC, Tom40, Sam50/Tob55, and Mdm10, were transferred to this complex and subsequently inserted into the OMM [25],[41]. Only recently, subunit Sam35 of the SAM/TOB complex was shown to recognize a specific sorting signal in the C-terminal part of mitochondrial β-barrel proteins and to initiate the insertion of these proteins into the OMM [42]. PorB is obviously lacking this sorting signal and is therefore not recognized as a substrate by the SAM complex. Interestingly, C-terminal sequences were also reported to be relevant in the sorting of β-barrel proteins in bacteria [43]. Our first attempt at directing PorB to the OMM by introducing a C-terminal segment of VDAC failed (data not shown), probably due to requirements in the tertiary structure of the protein, as shown previously for PorB [6].

Purified PorB spontaneously integrated into liposomes consisting of a broad variety of different lipids, from both the outer and inner mitochondrial membranes. Insertion of PorB into the lipid phase is accompanied by specific conformational changes (see Protocol S1 and Fig. S7) and requires neither additional assembly factors nor energy sources. A similar finding has been reported for the integration of soluble human VDAC into lipid bilayers [44]. Previously, we found significant amounts of PorB were associated with the OMM upon overexpression of the protein in yeast [6]; however, here we found that highly purified OMM vesicles are devoid of PorB. In HeLa cells, PorB shows the similar distribution pattern as the IMM protein Tim23. Interestingly, the secretin PulD, a β-barrel protein in the Gram-negative bacterium *Klebsiella oxytoca*, accumulates in the bacterial plasma membrane if assembly in the outer membrane is blocked [45]. We propose a similar mechanism for PorB: PorB trapped in the mitochondrial intermembrane space has the possibility to integrate into the IMM.

Since most proapoptotic factors of the mitochondria are stored in the IMS, recent investigations have concentrated on the question of how their release is triggered by the opening of the OMM. However, we found that during *Ngo*-induced apoptosis, events at the OMM are preceded by essential modifications at the IMM. Considering a typical size and a simplified volume to surface ratio, a single PorB channel would dissipate ΔΨ_m_ in about 0.8 ms (for details see Protocol S1). Thus, a single open PorB pore in the IMM is sufficient to short-circuit a whole mitochondrion. At potentials V_m_≥±60 mV, the PorB channel is enclosed in membranes with a lipid composition comparable to that of the IMM, but ATP binding arrests the channel in an open state even at voltages physiological for the IMM of about 150 mV. In previous experiments with time-averaged multichannel recordings, we observed an apparent ATP-dependent decrease in the voltage sensitivity of PorB channels [2]; however, the time-averaged analysis of these multichannel recordings did not allow the resolution of single-gating events. Here, our single-channel analyses unequivocally showed that ATP abolishes the voltage-dependent closure of PorB channels. In summary, following insertion into the IMM, PorB is arrested in an open state by ATP at the prevailing ΔΨ_m_, i.e. by the normal physiological activity of the mitochondria.

**Figure 8:**
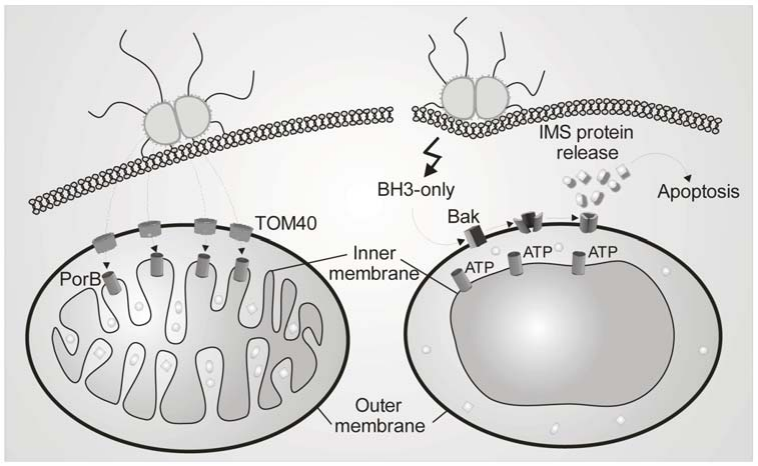
Model of PorB's role in infection induced apoptosis. PorB integrates into the inner membrane and causes an ATP-dependent loss of ΔΨ_m_. Cristae remodeling by PorB sensitizes mitochondria for the BH3-protein induced opening of the OMM by Bak.

The attenuated phenotype of the neisserial strain N886 expressing PorB*_K98Q_* provided clear *in vivo* evidence for a crucial role of ATP binding in dissipating ΔΨ_m_ and inducing apoptosis during infection. Our recent data suggest that cell death induced by Ngo requires the close interplay of two interdependent signaling cascades, one leading to the activation of Bak [11] and the other to the sensitization of the mitochondria by translocated PorB (Fig. 8). For the latter, PorB utilizes an existing mitochondrial transport pathway, the TOM complex, but bypasses the SAM complex, and activated by ATP dissipates ΔΨ_m_. This intriguing and so far unique example is a consequence of the evolution of the SAM machinery. SAM has diverted so much from the parental Omp85 machinery that it is now unable to recognize and sort PorB into the OMM. In this way the bacterial effector PorB functions as an integral element of the host's cell death machinery.

## Materials and Methods

### Bacterial strains and human cells

*N. gonorrhoeae* N242 (strain VPI; Opa+, PorB_IA_) [46] and N920 (strain MS11; Opa+, PorB_IA_) expressing PorB of N242 [35] have been described. N886 (strain MS11; Opa+, PorB*_K98Q_*) was constructed using the same strategy as for N920 but a mutant *porB* gene of N242 was transformed instead of the wildtype derivative [35]. Gonococci were routinely grown on GC agar base plates (Becton Dickinson, Difco and Remel) supplemented with Proteose Pepton Nr. 3 (Difco) and 1% vitamin mix for 14–20 h at 37°C in 5% CO_2_ in a humidified atmosphere. Opa phenotypes were monitored by colony morphology under a stereo microscope or by immunoblotting. HeLa cells (human cervix carcinoma) were grown in RPMI 1640 (Gibco) supplemented with 10% heat inactivated fetal calf serum (FCS) in the presence of 5% CO_2_. Cells were seeded 24 h before infection and washed several times with RPMI without supplements. Infections were routinely performed at a multiplicity of infection (MOI) of 1 without centrifugation. For inhibition of caspases, cells were incubated with 50 µM zVAD-fmk (Bachem) for 15 min prior to infection and throughout the respective infection period.

### Immunofluorescence microscopy

Cells were seeded on coverslips and transfected with pCMV-Tag-1, containing either the *porB_Ngo_* (P.IA) or *porB_Nmu_* gene with an N-terminal FLAG-Tag [3], using Lipofectamine 2000 (Invitrogen) according to the manufacturer's protocol. Twenty four hours post-transfection, cells were stained with 150 nM MitoTracker (Molecular Probes), dissolved in cell culture media for 30 min at 37°C, washed with phosphate buffered saline (PBS) and fixed in 3.7% paraformaldehyde (PFA). Fixed cells were permeabilized using 0.2% Triton X-100, and nonspecific binding was blocked by using 1% goat serum. Samples were stained using anti-FLAG (Sigma) antibody, followed by detection with fluorochrome-coupled secondary antibodies (Jackson Immuno Research). Samples were analyzed under a Leica confocal microscope using TCS software.

### Western blot

5×10^5^ cells per sample were harvested in 100 µl loading buffer and 20 µl of the protein lysates were separated by SDS-PAGE and transferred to nitrocellulose or polyvinylidenfluorid (PVDF) membranes. The following antibodies and sera were used in this study: anti-β-Actin (Sigma); anti-Bak NT (Upstate); anti-Bak (Ab-1) (Millipore); anti-Bax NT (Upstate); anti-Bax (6A7) (BD Pharmingen); anti-cleaved Caspase-3 (Cell Signalling); anti-FLAG (Sigma); anti-Hsp60 (Stressgen Bioreagents); anti-VDAC (Abcam); anti-Tim23 (BD Biosciences); antibodies against human Tom40 and Sam50 were a gift from N. J. Hoogenraad, and against mouse Metaxin 2 (cross-reactive with human Metaxin 2) a gift from P. Bornstein. Antibodies against yeast mitochondrial proteins Tom40 and Tim23 were a gift from N. Pfanner and C. Meisinger. Equal loading was routinely confirmed by appropriate loading controls. Quantitative analysis of immunoblots was performed by using the open source software ImageJ (http://rsbweb.nih.gov/ij/index.html).

### Mammalian mitochondria experiments

Trypsin treatment of mitochondria was performed as previously described [47]. To induce the knockdown by RNA interference, cells were grown for 5 to 7 days in the presence of 1 µg/ml doxycycline as previously described [27]. Efficiency of the knockdown was assessed by western blot. For trancription/translation purposes, PorB was cloned into pGEM-4Z vector (Promega) with two additional methionines at its C-terminus and *in vitro* transcribed/translated in the presence of ^35^S-methionine/cysteine (GE Healthcare) using the TnT Quick Coupled System (Promega). Mitochondrial isolation and import of proteins were performed essentially as described previously [27]. A detailed protocol is available as Protocol S1.

### Yeast mitochondria experiments

Mitochondria were isolated from yeast cells as described previously and used for import of ^35^S-labelled mitochondrial porin (VDAC), dicarboxylate carrier (DIC), or the ATP-synthase γ-subunit (F1γ) following standard procedures [15]. A detailed protocol is available as Protocol S1.

### Electrophysiological setup and measurements

Purified azolectin (60 mg/ml) in n-decan or a lipid mixture corresponding to the lipid composition of inner mitochondrial membranes (60 m/ml) [32] in n-decan was used to produce stable planar lipid bilayers by using the painting technique [48],[49]. Purified PorB was applied directly below the bilayer in the *cis* chamber. Buffer conditions were symmetrical with 250 mM KCl, 10 mM Mops-Tris (pH 7.0) in the *cis*/*trans* compartment. Two Ag/AgCl electrodes covered by 2 M KCl-agar bridges were inserted into each chamber with the *trans* chamber electrode connected to the headstage (CV-5-1GU) of a Geneclamp 500 current amplifier (Axon Instruments) and this was used as a reference for reported membrane potentials. Current recordings were carried out using a Digidata 1200 A/D converter. Data analysis was performed by self-written Windows-based SCIP (single-channel investigation program) in combination with Origin 7.0 (Microcal Software). Current recordings were performed at a sampling interval of 0.1 ms, filtered with a low-pass-filter at 2 kHz. Voltage ramps were carried out by continuously increasing the voltage at a rate of 5 mV/s.

### Nucleotide experiments

After incorporation of single PorB channels into the bilayer by spontaneous insertion, control currents were recorded. Subsequently nucleotides were added either to both sides or separately to the *cis* or *trans* side of the membrane. Interactions between the added nucleotides and PorB channels were examined after stirring the aqueous solutions on both sides of the planar lipid bilayer. Binding of ATP by PorB was assayed by chemically crosslinking radiolabelled ATP to neisserial strains expressing different PorB derivatives as previously described [2]. ATP binding was quantified using AIDA Image Analyzer software.

### Transmission electron microscopy

For immuno-EM analysis, the cells were fixed with 3% PFA in stabilizing buffer (1 mM EGTA, 4% PEG 6000 or PEG 8000, 100 mM PIPES pH 6.9) and embedded in 10% Gelatine/PBS. Small blocks of the samples were infiltrated overnight in 2.3 M sucrose/0.1 M Na-phosphate buffer. Ultra-thin sections were cut at −120°C with a diamond knife. The sections were transferred onto carbon-coated pioloform-film on TEM-grids. The sections were then blocked and reacted with the primary antibody against FLAG-tag (Sigma), Tom22 (GeneTex), Tom20 (BD Biosciences), Tim23 (BD Transduction Laboratories) and secondary antibodies coupled with 6 or 12 nm gold particles.

### FACS analysis

For the analysis of mitochondrial membrane potential cells were harvested by trypsinization and washed with phosphate buffered saline (PBS) before staining with 100 nM tetramethylrhodamine ethyl ester perchlorate (TMRE) (Molecular Probes) in growth media at 37°C, 5% CO_2_ for 30 min. After staining, cells were washed twice with PBS and immediately analyzed by FACS analysis.

## Supporting Information

Protocol S1 Supplemental Information.
(0.09 MB PDF)

Figure S1 (A) Isolated HeLa mitochondria (50 µg of protein) were incubated with radiolabelled PorB and subjected to carbonate extraction with 100 mM Na_2_CO_3_ at pH 10.8 or pH 11.5. Samples were analyzed by SDS-PAGE and western blot (Hsp60 and VDAC) or autoradiography (PorB). (B) Radiolabelled PorB was incubated with isolated mitochondria for the indicated times. Mock control represents the sample in which no mitochondria were present. All samples were subsequently subjected to carbonate extraction at pH 11.5 and analyzed by SDS-PAGE. The graph represents quantities of carbonate resistant PorB, where the 30 min time point was set to 100%. (C,D) Import of ^35^S-labelled PorB into yeast mitochondria. PorB was synthesized in reticulocyte lysate, incubated with mitochondria at 25°C for 10 min, and subsequently treated with proteinase K (PK) as described in the Materials and Methods section. As indicated, one sample was left without PK or without mitochondria, respectively. PorB was derived from *N. gonorrhoeae* (PorB*_Ngo_*) (C) or *N. mucosa* (PorB*_Nmu_*) (D), respectively. (E) Import of PorB into mitochondria from the yeast mutant strain *tom40-4* and from the corresponding wildtype strain. ^35^S-labelled PorB was synthesized in reticulocyte lysate and incubated with the mitochondria at 25°C for different times as indicated. The mitochondria were subsequently treated with PK at 0°C, reisolated, and analyzed by SDSPAGE and phosphorimager.
(1.37 MB TIF)

Figure S2 (A,B) Mitochondria from *sam50kd-2* and *mtx2kd-2* cell lines from the same fraction as were used for import of PorB were incubated with radiolabelled VDAC precursor for indicated times and then subjected to 50 µg/ml proteinase K treatment. The longest time point of the import into mitochondria from cells in which knockdowns were not induced by Dox was set to be 100%. (C,E) Import of ^35^S-labelled yeast VDAC (mitochondrial porin) into mitochondria isolated from the yeast mutant strain *sam50-1*, *Δsam37* yeast strain and from the corresponding wildtype strains. The relative amounts of imported porin were determined using a phosphorimager. The value of the longest time point for the import into wildtype strain was set to 100%. (D,F) Import of dicarboxylate carrier (DIC) in the absence of functional Sam50 and Sam37. The radiolabelled protein was synthesized in reticulocyte lysate and incubated with mitochondria isolated from indicated strains. At different time points samples were removed and treated with proteinase K.
(0.95 MB TIF)

Figure S3 PorB expressed in yeast is found in the mitoplast fraction after fractionation of mitochondria. PorB was expressed in yeast under the control of a Gal-inducible promoter. Mitochondrial outer membrane was isolated essentially as described in Zahedi et al. [12]. Outer membrane (Tom40) and inner membrane (Tim23) markers and PorB were detected by western blot. Shown is the mitochondrial fraction (Mitoch.) before separation of the outer membrane, the purified outer membrane vesicles (OMV) and the mitoplast fraction (MPF). Note that PorB is absent from the highly purified OMV but co-purifies with the mitoplast fraction.
(0.78 MB TIF)

Figure S4 Current-voltage relationship of PorB. The main conductance (blue) and the two most frequent subconductance states (red and black) are shown. Insert shows a conductance histogram calculated from some 10,000 gating events of PorB.
(0.76 MB TIF)

Figure S5 HeLa cells infected with strain N920 in the absence or presence of caspase inhibitor zVAD were loaded with calcein-AM (green) and CoCl_2_ as mentioned in Protocol S1. Cells were then incubated with MitoTracker Orange (red) to check for membrane potential loss. The respective overlays are shown.
(2.39 MB TIF)

Figure S6 PorB_K98Q_ is targeted to mitochondria upon overexpression. HeLa cells were transfected with expression constructs for wildtype PorB (PorB*_Ngo_*) and ATP-binding mutant (PorB*_K98Q_*) at two different concentrations (0.25 and 0.5 µg/ml) and the expression of PorB (FLAG) and the presence of ΔΨ_m_ (MitoTracker) were monitored.
(2.56 MB TIF)

Figure S7 (A) An assessment of the secondary structure of PorB. CD spectra of PorB in solution (20 mM K_2_HPO_4_/KH_2_PO_4_) and of renatured PorB in decylmaltoside. (B) CD spectra of PorB in solution (20 mM K_2_HPO4/KH_2_PO_4_) and of renatured PorB in liposomes. Experiments were performed as described [13].
(0.78 MB TIF)

## References

[1] Weel JF, van Putten JP (1991). Fate of the major outer membrane protein P.IA in early and late events of gonococcal infection of epithelial cells.. Res Microbiol.

[2] Rudel T, Schmid A, Benz R, Kolb HA, Lang F (1996). Modulation of Neisseria porin (PorB) by cytosolic ATP/GTP of target cells: parallels between pathogen accommodation and mitochondrial endosymbiosis.. Cell.

[3] Müller A, Gunther D, Brinkmann V, Hurwitz R, Meyer TF (2000). Targeting of the pro-apoptotic VDAC-like porin (PorB) of Neisseria gonorrhoeae to mitochondria of infected cells.. EMBO J.

[4] Massari P, Ho Y, Wetzler LM (2000). Neisseria meningitidis porin PorB interacts with mitochondria and protects cells from apoptosis.. Proc Natl Acad Sci U S A.

[5] Kroemer G, Reed JC (2000). Mitrochondrial control of cell death.. Nature Med.

[6] Müller A, Rassow J, Grimm J, Machuy N, Meyer TF (2002). VDAC and the bacterial porin PorB of Neisseria gonorrhoeae share mitochondrial import pathways.. EMBO J.

[7] Chipuk JE, Green DR (2008). How do BCL-2 proteins induce mitochondrial outer membrane permeabilization?. Trends Cell Biol.

[8] Desagher S, Osen-Sand A, Nichols A, Eskes R, Montessuit S (1999). Bid-induced conformational change of Bax is responsible for mitochondrial cytochrome c release during apoptosis.. J Cell Biol.

[9] Wei MC, Lindsten T, Mootha VK, Weiler S, Gross A (2000). tBID, a membrane-targeted death ligand, oligomerizes BAK to release cytochrome c.. Genes Dev.

[10] Kepp O, Gottschalk K, Churin Y, Rajalingam K, Brinkmann V (2009). Bim and Bmf synergize to induce apoptosis in Neisseria gonorrhoeae infection.. PLoS Pathog.

[11] Kepp O, Rajalingam K, Kimmig S, Rudel T (2007). Bak and Bax are non-redundant during infection- and DNA damage-induced apoptosis.. EMBO J.

[12] Walther DM, Papic D, Bos MP, Tommassen J, Rapaport D (2009). Signals in bacterial beta-barrel proteins are functional in eukaryotic cells for targeting to and assembly in mitochondria.. Proc Natl Acad Sci U S A.

[13] Pfanner N, Douglas MG, Endo T, Hoogenraad NJ, Jensen RE (1996). Uniform nomenclature for the protein transport machinery of the mitochondrial membranes.. Trends Biochem Sci.

[14] Hill K, Model K, Ryan MT, Dietmeier K, Martin F (1998). Tom40 forms the hydrophilic channel of the mitochondrial import pore for preproteins [see comment].. Nature.

[15] Krimmer T, Rapaport D, Ryan MT, Meisinger C, Kassenbrock (2001). Biogenesis of porin of the outer mitochondrial membrane involves an import pathway via receptors and the general import pore of the TOM complex.. J Cell Biol.

[16] Wiedemann N, Kozjak V, Chacinska A, Schonfisch B, Rospert S (2003). Machinery for protein sorting and assembly in the mitochondrial outer membrane.. Nature.

[17] Paschen SA, Waizenegger T, Stan T, Preuss M, Cyrklaff M (2003). Evolutionary conservation of biogenesis of beta-barrel membrane proteins.. Nature.

[18] Gentle IE, Burri L, Lithgow T (2005). Molecular architecture and function of the Omp85 family of proteins.. Molecular Microbiology.

[19] Voulhoux R, Bos MP, Geurtsen J, Mols M, Tommassen J (2003). Role of a highly conserved bacterial protein in outer membrane protein assembly.. Science.

[20] Bos MP, Tommassen J (2004). Biogenesis of the Gram-negative bacterial outer membrane.. Current Opinion in Microbiology.

[21] Degterev A, Lugovskoy A, Cardone M, Mulley B, Wagner G (2001). Identification of small-molecule inhibitors of interaction between the BH3 domain and Bcl-xL.. Nat Cell Biol.

[22] Scorrano L, Ashiya M, Buttle K, Weiler S, Oakes SA (2002). A distinct pathway remodels mitochondrial cristae and mobilizes cytochrome c during apoptosis.. Dev Cell.

[23] Gottlieb E, Armour SM, Thompson CB (2002). Mitochondrial respiratory control is lost during growth factor deprivation.. Proc Natl Acad Sci U S A.

[24] Gottlieb E, Armour SM, Harris MH, Thompson CB (2003). Mitochondrial membrane potential regulates matrix configuration and cytochrome c release during apoptosis.. Cell Death Differ.

[25] Paschen SA, Neupert W, Rapaport D (2005). Biogenesis of beta-barrel membrane proteins of mitochondria.. Trends Biochem Sci.

[26] Bolender N, Sickmann A, Wagner R, Meisinger C, Pfanner N (2008). Multiple pathways for sorting mitochondrial precursor proteins.. EMBO Rep.

[27] Kozjak-Pavlovic V, Ross K, Benlasfer N, Kimmig S, Karlas A (2007). Conserved roles of Sam50 and metaxins in VDAC biogenesis.. EMBO Rep.

[28] Kozjak V, Wiedemann N, Milenkovic D, Lohaus C, Meyer HE (2003). An essential role of Sam50 in the protein sorting and assembly machinery of the mitochondrial outer membrane.. J Biol Chem.

[29] Armstrong LC, Komiya T, Bergman BE, Mihara K, Bornstein P (1997). Metaxin is a component of a preprotein import complex in the outer membrane of the mammalian mitochondrion.. J Biol Chem.

[30] Zahedi RP, Sickmann A, Boehm AM, Winkler C, Zufall N (2006). Proteomic analysis of the yeast mitochondrial outer membrane reveals accumulation of a subclass of preproteins.. Mol Biol Cell.

[31] Song J, Minetti CA, Blake MS, Colombini M (1998). Successful recovery of the normal electrophysiological properties of PorB (class 3) porin from Neisseria meningitidis after expression in Escherichia coli and renaturation.. Biochim Biophys Acta.

[32] Voelker DR (2004). Lipid synthesis and transport in mitochondrial biogenisis.. Topics in Current Genetics.

[33] Loew LM, Tuft RA, Carrington W, Fay FS (1993). Imaging in five dimensions: time-dependent membrane potentials in individual mitochondria.. Biophys J.

[34] Castora FJ, Kelly WG (1986). ATP inhibits nuclear and mitochondrial type I topoisomerases from human leukemia cells.. Proc Natl Acad Sci U S A.

[35] Bauer FJ, Rudel T, Stein M, Meyer TF (1999). Mutagenesis of the Neisseria gonorrhoeae porin reduces invasion in epithelial cells and enhances phagocyte responsiveness.. Mol Microbiol.

[36] Massari P, King CA, Ho AY, Wetzler LM (2003). Neisserial PorB is translocated to the mitochondria of HeLa cells infected with Neisseria meningitidis and protects cells from apoptosis.. Cell Microbiol.

[37] Deghmane AE, Veckerle C, Giorgini D, Hong E, Ruckly C (2009). Differential modulation of TNF-alpha-induced apoptosis by Neisseria meningitidis.. PLoS Pathog.

[38] Neupert W, Brunner M (2002). The protein import motor of mitochondria.. Nat Rev Mol Cell Biol.

[39] Wiedemann N, Frazier AE, Pfanner N (2004). The protein import machinery of mitochondria.. J Biol Chem.

[40] Schatz G (1996). The protein import system of mitochondria.. J Biol Chem.

[41] Taylor RD, Pfanner N (2004). The protein import and assembly machinery of the mitochondrial outer membrane.. Biochim Biophys Acta.

[42] Kutik S, Stojanovski D, Becker L, Becker T, Meinecke M (2008). Dissecting membrane insertion of mitochondrial beta-barrel proteins.. Cell.

[43] Robert V, Volokhina EB, Senf F, Bos MP, Van Gelder P (2006). Assembly factor Omp85 recognizes its outer membrane protein substrates by a species-specific C-terminal motif.. PLoS Biol.

[44] Shanmugavadivu B, Apell HJ, Meins T, Zeth K, Kleinschmidt JH (2007). Correct folding of the beta-barrel of the human membrane protein VDAC requires a lipid bilayer.. J Mol Biol.

[45] Guilvout I, Chami M, Engel A, Pugsley AP, Bayan N (2006). Bacterial outer membrane secretin PulD assembles and inserts into the inner membrane in the absence of its pilotin.. EMBO J.

[46] Makino S, van Putten JP, Meyer TF (1991). Phase variation of the opacity outer membrane protein controls invasion by Neisseria gonorrhoeae into human epithelial cells.. EMBO J.

[47] Pon LA, Schon EA (2001). Mitochondria.

[48] Meinecke M, Wagner R, Kovermann P, Guiard B, Mick DU (2006). Tim50 maintains the permeability barrier of the mitochondrial inner membrane.. Science.

[49] Mueller P, Rudin DO, Tien HT, Wescott WC (1962). Reconstitution of cell membrane structure in vitro and its transformation into an excitable system.. Nature.

