# Supplementary material for: Bacterial Porin Disrupts Mitochondrial Membrane Potential and Sensitizes Host Cells to Apoptosis
Source: PLoS Pathog. 2009 Oct 23;5(10):e1000629. doi: 10.1371/journal.ppat.1000629 (PMC2759283; doi:10.1371/journal.ppat.1000629)
Supplement: Protocol S1 — Supplemental Information. (0.09 MB PDF) [file ppat.1000629.s001.pdf]

Supplemental Information

The secondary structures of PorB in liposomes and its soluble form are different

We compared the secondary structure of PorB in solution and in detergent solution with the corresponding structure of the protein in liposomes using circular dichroism (CD) spectroscopy (Fig. S7A,B). Spectra were analyzed with the CDpro package [1–3] using three different algorithms to fit the spectra and in addition with a neural network approach [4]. As expected [5] PorB reveals a significantly higher β-sheet content as compared to α-helical structures. Spectra of PorB in solution and in the presence of decylmaltoside (DM) showed similar shapes (Fig. S7A). However, the shape of the PorB CD spectrum in liposomes was significantly different from the one of the soluble form (Fig. S7B). Analysis of this spectrum showed a considerable increase of β-sheet structures and an almost complete loss of α-helical structures after incorporation of the protein into liposomes. Both CD spectra and calculations of the spectra clearly demonstrate the large structural changes of PorB during membrane insertion.

Calculation of membrane potential dissipation

The capacity of a biological membrane is the product of the specific capacity (for a typical biological membrane this can be estimated with $c_M = 1.0\ \mu F \cdot cm^{-2}$) and the surface. Considering a mitochondrion as a prolate with an axial ratio of a = c = 1/5 b and a length of b = 5 µm the membrane capacity can be estimated as follows:

The surface of the mitochondrion is:

$$A_M = \frac{2 \cdot \pi \cdot a^2 \cdot b}{\sqrt{a^2 - b^2}} \cdot \left[\frac{b}{a^2} \cdot \sqrt{a^2 - b^2} + ar\sin\left(\frac{\sqrt{a^2 - b^2}}{a} \right) \right]$$

$$A_M = 34.1 \mu m^2$$

Where A_M is the surface and a, b and c are the axes of the spheroid.

Thus the capacity (C) of a mitochondrion is:

$$C = c_M \cdot A_M$$

$$C = 0.34 pF$$

Next one has to estimate how many charges (Q) need to cross the membrane to build up a potential of V = 150 mV:

$$C = \frac{Q}{V}$$

$$Q = V \cdot C$$

$$Q = 5.11 \cdot 10^{-14} C$$

Under the simplified assumption that the potential is only build up by monovalent ions one can calculate how many ions (N_M) have to pass the membrane:

$$N = \frac{Q}{z \cdot F_{el}}$$

$$N = 5.3 \cdot 10^{-19} mol$$

$$N_M = N \cdot N_A$$

$$N_M = 3.19 \cdot 10^5$$

Where z is the valence of the ions and F_{el} is the faraday constant.

Having the conductance of the PorB channel with G = 420 pS and the membrane potential of V = 150 mV one can now calculate how many ions (N_P) pass the channel at physiological membrane potential per second:

$$G = \frac{I}{V}$$

$$I = G \cdot V$$

$$I = 63\,pA$$

$$N_P = \frac{I}{e_0}$$

$$N_P = 3.93 \cdot 10^8 \cdot s^{-1}$$

Where I is the current and e_0 is the elementary charge.

Dividing the number of ions needed to establish the membrane potential with the number of ions passing the channel one obtains the approximate time (t) in which one PorB channel could dissipate the mitochondrial inner membrane potential:

$$t = \frac{N_M}{N_P}$$

$$t = 0.8ms$$

Assessment of proton pump rates and leak currents

I) Proton pumping capacity of a mitochondrion

Measurements with rat heart mitochondria (*1*) and model calculations (*2*) show that the maximum rate of ATP production by a single mitochondrion is:

$$v_{\max} = 0.0133 \frac{fmol}{s}\, per\, mitochondrion\; or\; 8 \cdot 10^6\, molecules\; ATP / s\,.$$

Considering the now widely accepted H^+/ATP ratio of $10H^+$/3ATP (*3*) and assuming that the maximal ATP production occurs at a maximal H^+ pump rate we obtain :

$$v_{\max}^{H^+ pump} = 2{,}3 \cdot 10^7\, H^+ / s\;\; per\; mitochondrion$$

II) Turnover rate of Ngo PorB currents

The Ngo PorB channel revealed a conductance of G=420 pS for one of the three pore units. Assuming a steady state membrane potential of $\Delta\psi_m \cong 150$ mV (*4;5*) for respiring mitochondria we can under this conditions calculate the current flow through single Ngo PorB channel (N_p) in the IMM:

$$G = \frac{I}{U} \qquad I := G \cdot U \qquad I = 6.3 \times 10^{-11} A$$

I =current, U=voltage

e_0=elementary charge

$$N_P := \frac{I}{e_o} \qquad N_P = 3.932 \times 10^{8} \cdot s^{-1}$$ at $\Delta\psi_m$=150 mV,

$$N_P = 1.311 \times 10^{8} \cdot s^{-1}$$ at $\Delta\psi_m$=50 mV

Thus the maximal proton pump rate of a single mitochondrion and the leak currents through a single PorB channel pore are presumed to be in the same order of magnitude.

Materials and methods

Purification of PorB

Neisserial PorB porin was purified as described by Achtman et al., [8]. Briefly, an overnight culture of the respective Neisseria strain was harvested, washed and resuspended in buffer A (1 M NaAc pH 4, 1 mM PMSF). Bacteria were lysed in 9 volumes of buffer B (0.5% Zwittergent; 0.5 M $CaCl_2$; 1 mM PMSF). Non-soluble proteins were removed by centrifugation and ethanol was added to the supernatant at a final concentration of 20 % to precipitate surplus proteins. The supernatant was dialysed against buffer D (20 mM Tris pH 8; 2 mM $MgCl_2$; 0.05 % Zwittergent) and for further purification loaded on a SQ15 anion exchange column. Porin was eluted in a linear gradient from 0 to 1 M NaCl in buffer D. Fractions were analyzed for purity by SDS-PAGE, coomassie and silverstaining. Purified PorB fractions contained at least 70 % of the trimeric form.

Mammalian mitochondria experiments

Mitochondrial isolation and import of proteins were performed essentially as described previously [9]. Following the import, mitochondria were either treated with 50 µg/ml of proteinase K in the case of VDAC, or subjected to carbonate extraction. To this end, mitochondria (50 µg of protein) were solubilized in 100 µl of 100 mM Na_2CO_3, pH 11.5 (or pH 10.8 where indicated), incubated for 30 min on ice, and centrifuged for 30 min at 100 000 g. The pellet was then solubilized in Laemmli buffer and subjected to SDS-PAGE. Where supernatant was analyzed, proteins were first precipitated with trichloroacetic acid (TCA). Radiolabelled proteins were visualized using Fuji FLA3000 imaging system and the intensity of the bands was quantified using AIDA Image Analyzer software.

Yeast mitochondria experiments

Mitochondria of the temperature-sensitive strain *sam50-1* were preincubated for 15 min at 37°C to induce the mutant phenotype; the corresponding wildtype mitochondria were treated in the same way [10]. The radiolabelled proteins were synthesized in rabbit reticulocyte lysate (TNT, Promega) in the presence of ^{35}S-methionine (MP Biomedicals). Import of PorB was carried out following a modified protocol: reticulocyte lysate containing radiolabelled PorB was preincubated with 25 mM HCl for 10 min at 20°C. In parallel, isolated yeast mitochondria (20 µg protein) were preincubated for 5 min at 25°C in the presence of 4 µl non-radioactive reticulocyte lysate and BSA buffer (3% (w/v) BSA, 250 mM sucrose, 80 mM KCl, 1 mM NADH, 1 mM EDTA, 10 mM MOPS and 20 mM potassium phosphate pH 7.4) in a total volume of 23 µl. Eventually, 2 µl acid-treated reticulocyte lysate were added to the suspension of mitochondria and the samples were incubated at 25°C (usually for 5 min) to allow binding of PorB. The samples were subsequently cooled on ice, the mixture was layered on top of a 500 µl sucrose cushion containing 500 mM sucrose, 1 mM EDTA and 10 mM MOPS, pH 7.4, and the mitochondria were reisolated by centrifugation for 10 min at 23.000 g. The mitochondria were then resuspended in BSA buffer containing 5 mM $MgCl_2$ and 1 mM ATP instead of EDTA and incubated at 25°C to allow translocation of PorB across the mitochondrial outer membrane. The samples were eventually treated with 50 µg/ml proteinase K for 10 min on ice, proteolysis was stopped by addition of 4 mM phenylmethylsulphonyl fluoride (PMSF), and the mitochondria were reisolated for analysis by SDS-PAGE and digital autoradiography. Mitochondrial inner and outer membrane vesicles were separated according to Kozjak et al. [10].

Calcein quenching assay

Calcein-AM when added to cells is hydrolyzed by intraorganellar esterases, yielding calcein, which is hydrophilic and therefore trapped inside the intracellular organelles. If the calcein loaded cells are treated with cobalt chloride ($CoCl_2$) the calcein-dependent fluorescence is quenched except in the mitochondrial matrix, as $CoCl_2$ cannot penetrate the mitochondrial inner membrane. Permeabilization of the inner membrane leads to the exposure of calcein to $CoCl_2$ and thereby to the loss of green fluorescence. To measure the perforation of the mitochondrial inner membrane by calcein-AM release experiments the cells were stained with 5 µM calcein-AM and 1 mM $CoCl_2$ in growth media for 15 min at 37°C, 5% CO_2 and washed twice with PBS prior to MitoTracker staining and fixation.

Circular dichroism-spectroscopy

Purified proteins were analyzed under three different conditions. PorB in solution: the protein was dialyzed over night at 4°C against a buffer containing 20 mM K_2HPO_4/KH_2PO_4, 10 mM KCl, pH 7.0. PorB solubilized in detergent solution: the protein was dialyzed against the same P_i-buffer in addition with 8 mM N-Decyl-β-D-Maltopyranosid. To insert PorB into membrane vesicles, small azolectin-liposomes were formed as described [11]. Liposomes were incubated with purified protein for 2.5 h at room temperature and subsequently dialyzed over night at 4°C against 20 mM K_2HPO_4/KH_2PO_4, 10 mM KCl, pH 7.0. All three samples were adjusted to the protein concentration of 550 µg / ml.

CD spectra were recorded using a Jasco J-810 spectrapolarimeter. All measurements were carried out in a quartz cuvette with an optical path length of 0.01 cm at room temperature. The scans (n = 16) were averaged to improve the signal / noise ratio. Blank buffer spectra were collected and subtracted from the sample spectra. To compare the

different data sets they were converted to mean residue ellipticity and subsequently analyzed using the CDpro package [1–3] and a neural network approach [4].

References

1. Sreerama N, Woody RW (2000) Estimation of protein secondary structure from circular dichroism spectra: comparison of CONTIN, SELCON, and CDSSTR methods with an expanded reference set. Anal Biochem 287: 252-260.

2. Sreerama N, Woody RW (2003) Structural composition of betaI- and betaII-proteins. Protein Sci 12: 384-388.

3. Sreerama N, Woody RW (2004) On the analysis of membrane protein circular dichroism spectra. Protein Sci 13: 100-112.

4. Bohm G, Muhr R, Jaenicke R (1992) Quantitative analysis of protein far UV circular dichroism spectra by neural networks. Protein Eng 5: 191-195.

5. Derrick JP, Urwin R, Suker J, Feavers IM, Maiden MC (1999) Structural and evolutionary inference from molecular variation in Neisseria porins. Infect Immun 67: 2406-2413.

6. Makino S, van Putten JP, Meyer TF (1991) Phase variation of the opacity outer membrane protein controls invasion by Neisseria gonorrhoeae into human epithelial cells. EMBO J 10: 1307-1315.

7. Bauer FJ, Rudel T, Stein M, Meyer TF (1999) Mutagenesis of the Neisseria gonorrhoeae porin reduces invasion in epithelial cells and enhances phagocyte responsiveness. Mol Microbiol 31: 903-913.

8. Achtman M, Neibert M, Crowe BA, Strittmatter W, Kusecek B et al. (1988) Purification and characterization of eight class 5 outer membrane protein variants from a clone of Neisseria meningitidis serogroup A. J Exp Med 168: 507-525.

9. Kozjak-Pavlovic V, Ross K, Benlasfer N, Kimmig S, Karlas A et al. (2007) Conserved roles of Sam50 and metaxins in VDAC biogenesis. EMBO Rep 8: 576-582.

10. Kozjak V, Wiedemann N, Milenkovic D, Lohaus C, Meyer HE et al. (2003) An essential role of Sam50 in the protein sorting and assembly machinery of the mitochondrial outer membrane. J Biol Chem 278: 48520-48523.

11. Meinecke M, Wagner R, Kovermann P, Guiard B, Mick DU et al. (2006) Tim50 maintains the permeability barrier of the mitochondrial inner membrane. Science 312: 1523-1526.

12. Zahedi RP, Sickmann A, Boehm AM, Winkler C, Zufall N et al. (2006) Proteomic analysis of the yeast mitochondrial outer membrane reveals accumulation of a subclass of preproteins. Mol Biol Cell 17: 1436-1450.

13. Becker L, Bannwarth M, Meisinger C, Hill K, Model K et al. (2005) Preprotein translocase of the outer mitochondrial membrane: reconstituted Tom40 forms a characteristic TOM pore. J Mol Biol 353: 1011-1020.
