# Supplementary figures and images for: Bacterial Porin Disrupts Mitochondrial Membrane Potential and Sensitizes Host Cells to Apoptosis
Source: PLoS Pathog. 2009 Oct 23;5(10):e1000629. doi: 10.1371/journal.ppat.1000629 (PMC2759283; doi:10.1371/journal.ppat.1000629)

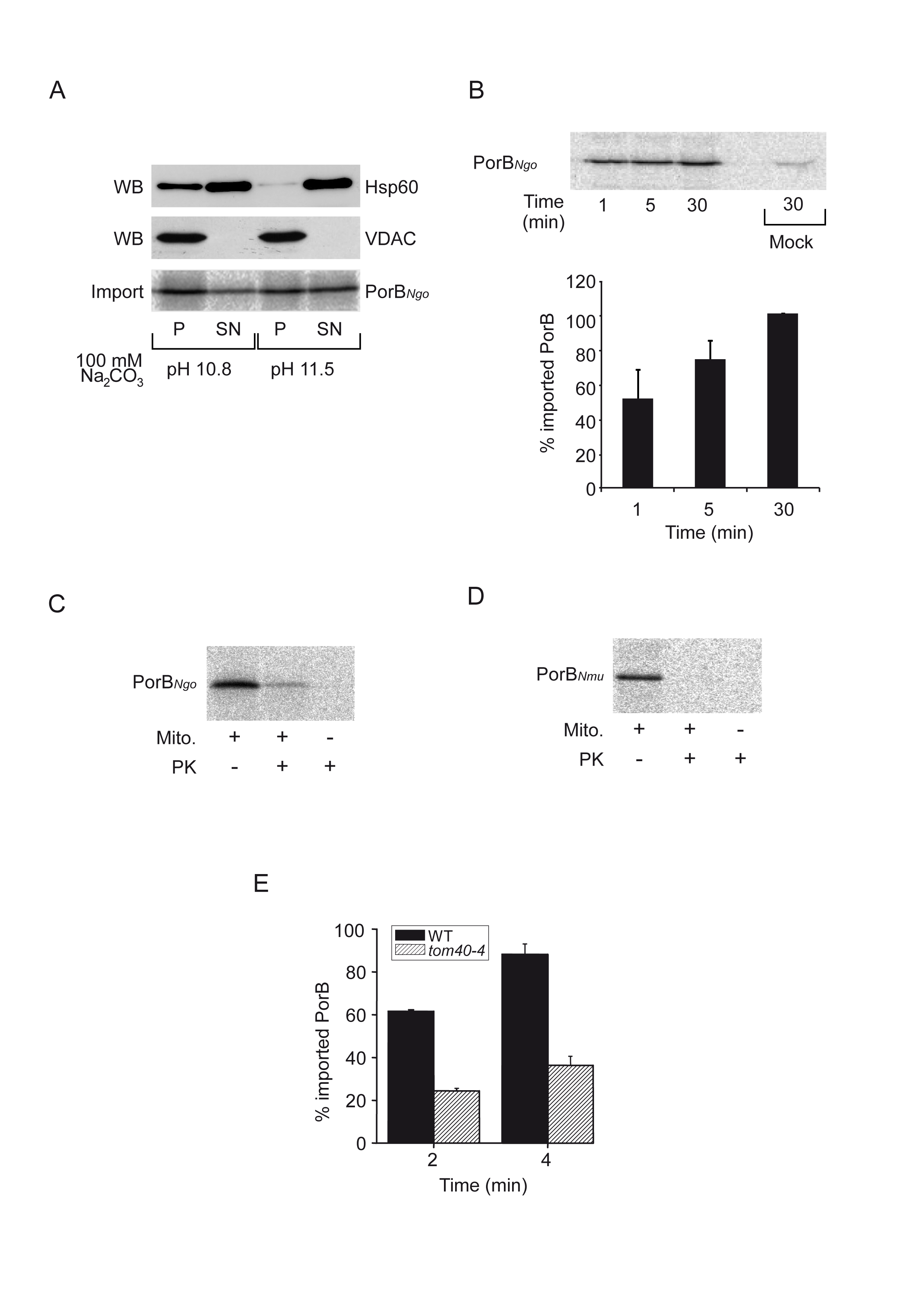

Supplement: Figure S1 — (A) Isolated HeLa mitochondria (50 µg of protein) were incubated with radiolabelled PorB and subjected to carbonate extraction with 100 mM Na2CO3 at pH 10.8 or pH 11.5. Samples were analyzed by SDS-PAGE and western blot (Hsp60 and VDAC) or autoradiography (PorB). (B) Radiolabelled PorB was incubated with isolated mitochondria for the indicated times. Mock control represents the sample in which no mitochondria were present. All samples were subsequently subjected to carbonate extraction at pH 11.5 and analyzed by SDS-PAGE. The graph represents quantities of carbonate resistant PorB, where the 30 min time point was set to 100%. (C,D) Import of 35S-labelled PorB into yeast mitochondria. PorB was synthesized in reticulocyte lysate, incubated with mitochondria at 25°C for 10 min, and subsequently treated with proteinase K (PK) as described in the Materials and Methods section. As indicated, one sample was left without PK or without mitochondria, respectively. PorB was derived from N. gonorrhoeae (PorBNgo) (C) or N. mucosa (PorBNmu) (D), respectively. (E) Import of PorB into mitochondria from the yeast mutant strain tom40-4 and from the corresponding wildtype strain. 35S-labelled PorB was synthesized in reticulocyte lysate and incubated with the mitochondria at 25°C for different times as indicated. The mitochondria were subsequently treated with PK at 0°C, reisolated, and analyzed by SDSPAGE and phosphorimager. (1.37 MB TIF) [file ppat.1000629.s002.tif]

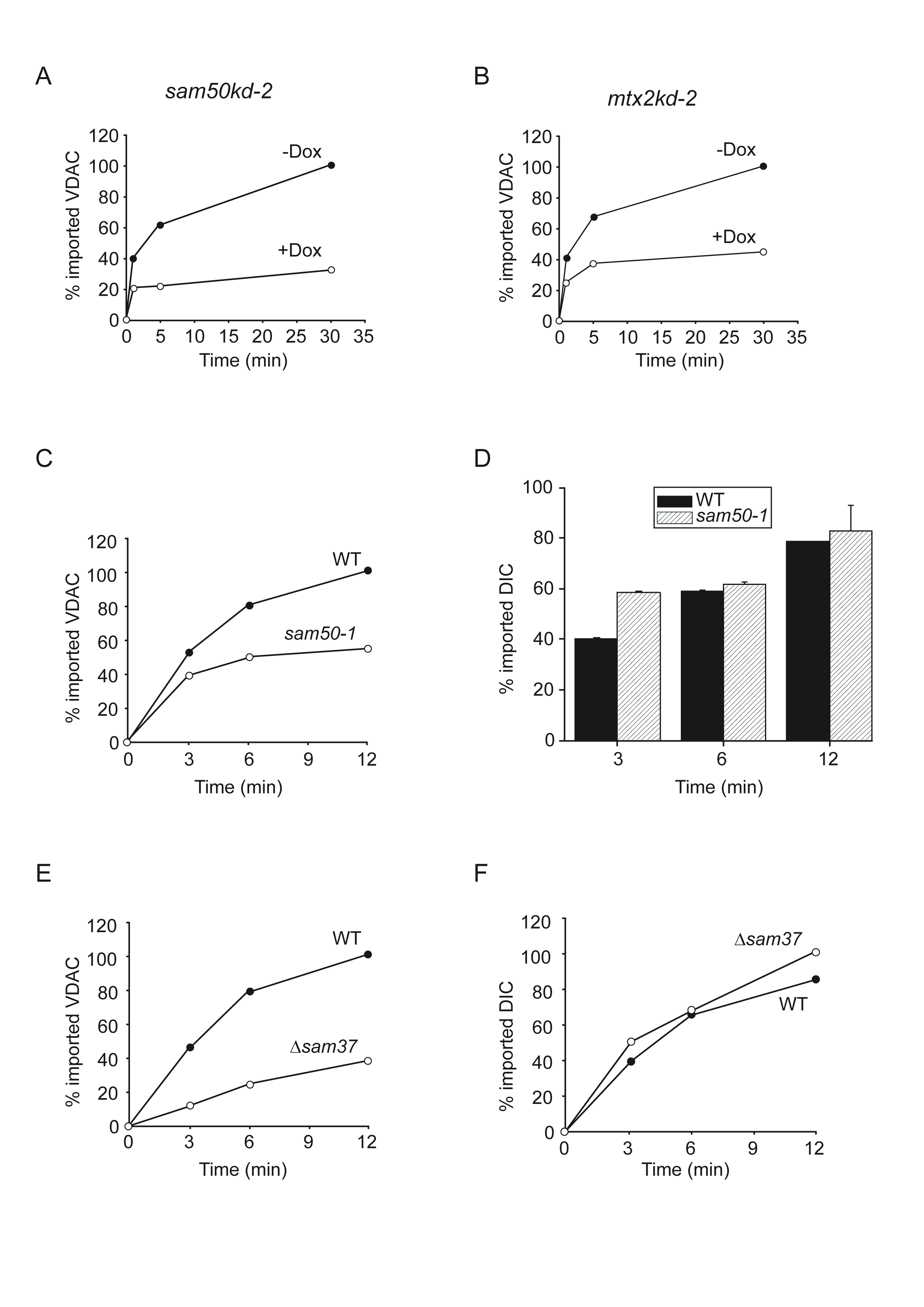

Supplement: Figure S2 — (A,B) Mitochondria from sam50kd-2 and mtx2kd-2 cell lines from the same fraction as were used for import of PorB were incubated with radiolabelled VDAC precursor for indicated times and then subjected to 50 µg/ml proteinase K treatment. The longest time point of the import into mitochondria from cells in which knockdowns were not induced by Dox was set to be 100%. (C,E) Import of 35S-labelled yeast VDAC (mitochondrial porin) into mitochondria isolated from the yeast mutant strain sam50-1, Δsam37 yeast strain and from the corresponding wildtype strains. The relative amounts of imported porin were determined using a phosphorimager. The value of the longest time point for the import into wildtype strain was set to 100%. (D,F) Import of dicarboxylate carrier (DIC) in the absence of functional Sam50 and Sam37. The radiolabelled protein was synthesized in reticulocyte lysate and incubated with mitochondria isolated from indicated strains. At different time points samples were removed and treated with proteinase K. (0.95 MB TIF) [file ppat.1000629.s003.tif]

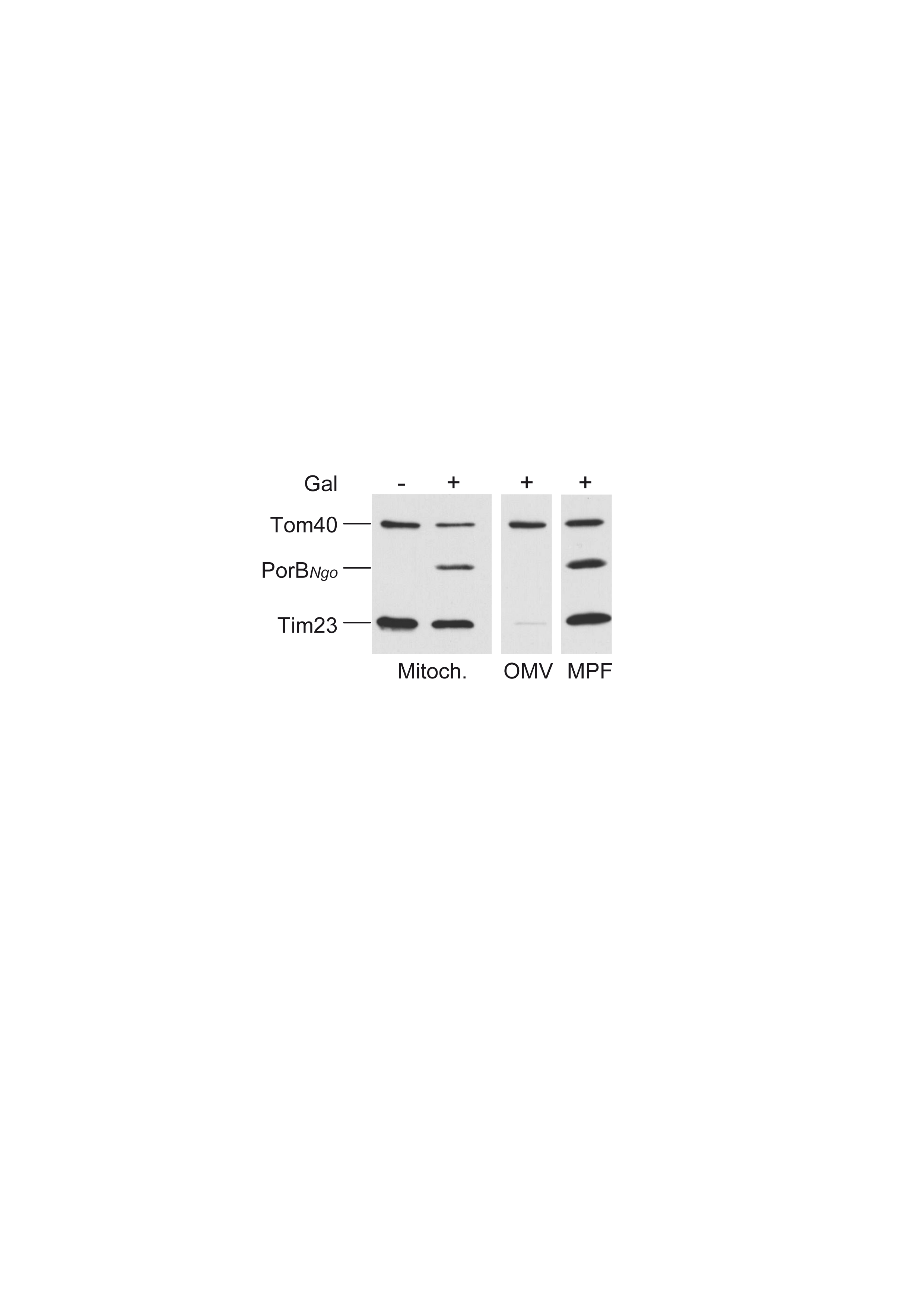

Supplement: Figure S3 — PorB expressed in yeast is found in the mitoplast fraction after fractionation of mitochondria. PorB was expressed in yeast under the control of a Gal-inducible promoter. Mitochondrial outer membrane was isolated essentially as described in Zahedi et al. [12]. Outer membrane (Tom40) and inner membrane (Tim23) markers and PorB were detected by western blot. Shown is the mitochondrial fraction (Mitoch.) before separation of the outer membrane, the purified outer membrane vesicles (OMV) and the mitoplast fraction (MPF). Note that PorB is absent from the highly purified OMV but co-purifies with the mitoplast fraction. (0.78 MB TIF) [file ppat.1000629.s004.tif]

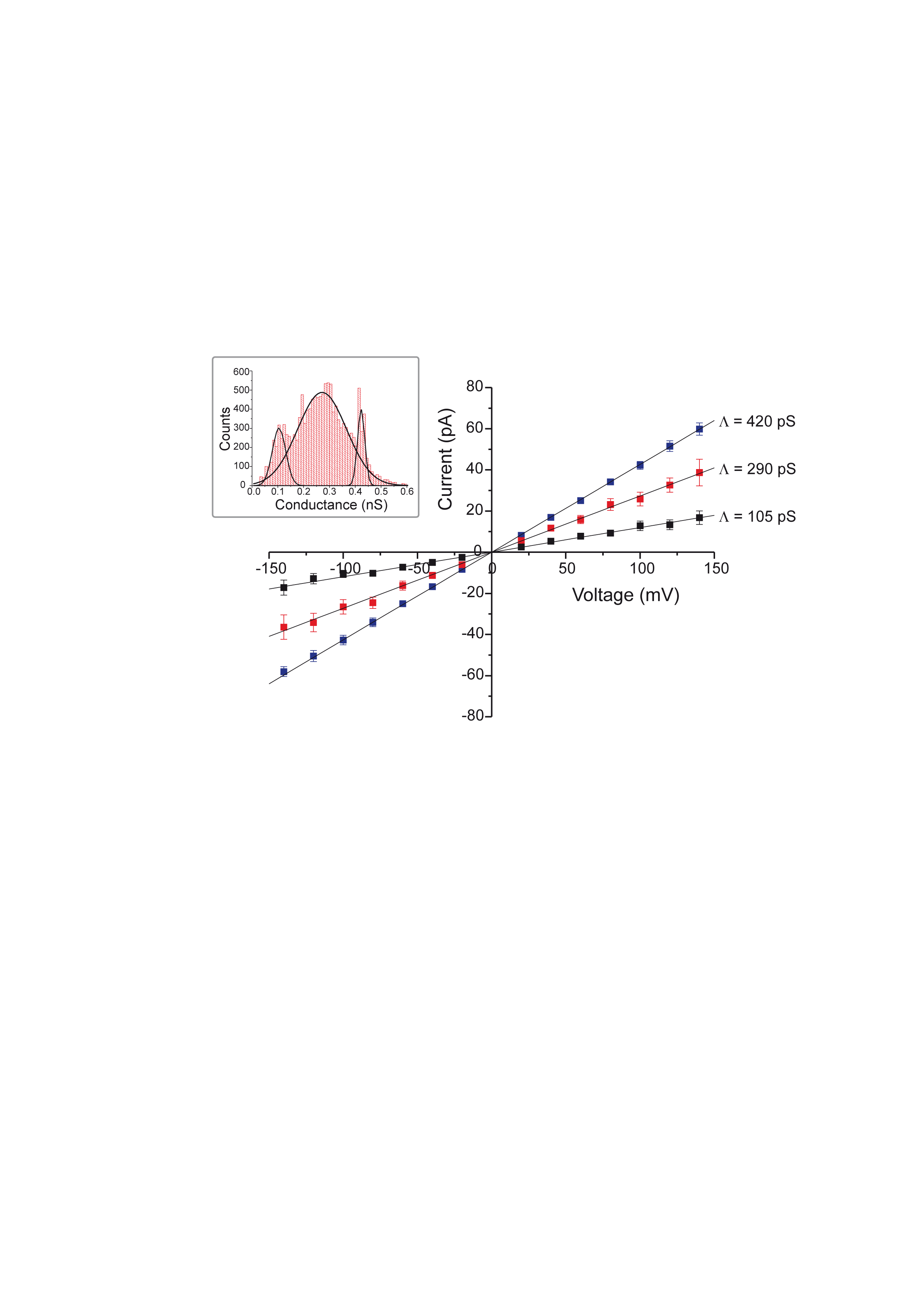

Supplement: Figure S4 — Current-voltage relationship of PorB. The main conductance (blue) and the two most frequent subconductance states (red and black) are shown. Insert shows a conductance histogram calculated from some 10,000 gating events of PorB. (0.76 MB TIF) [file ppat.1000629.s005.tif]

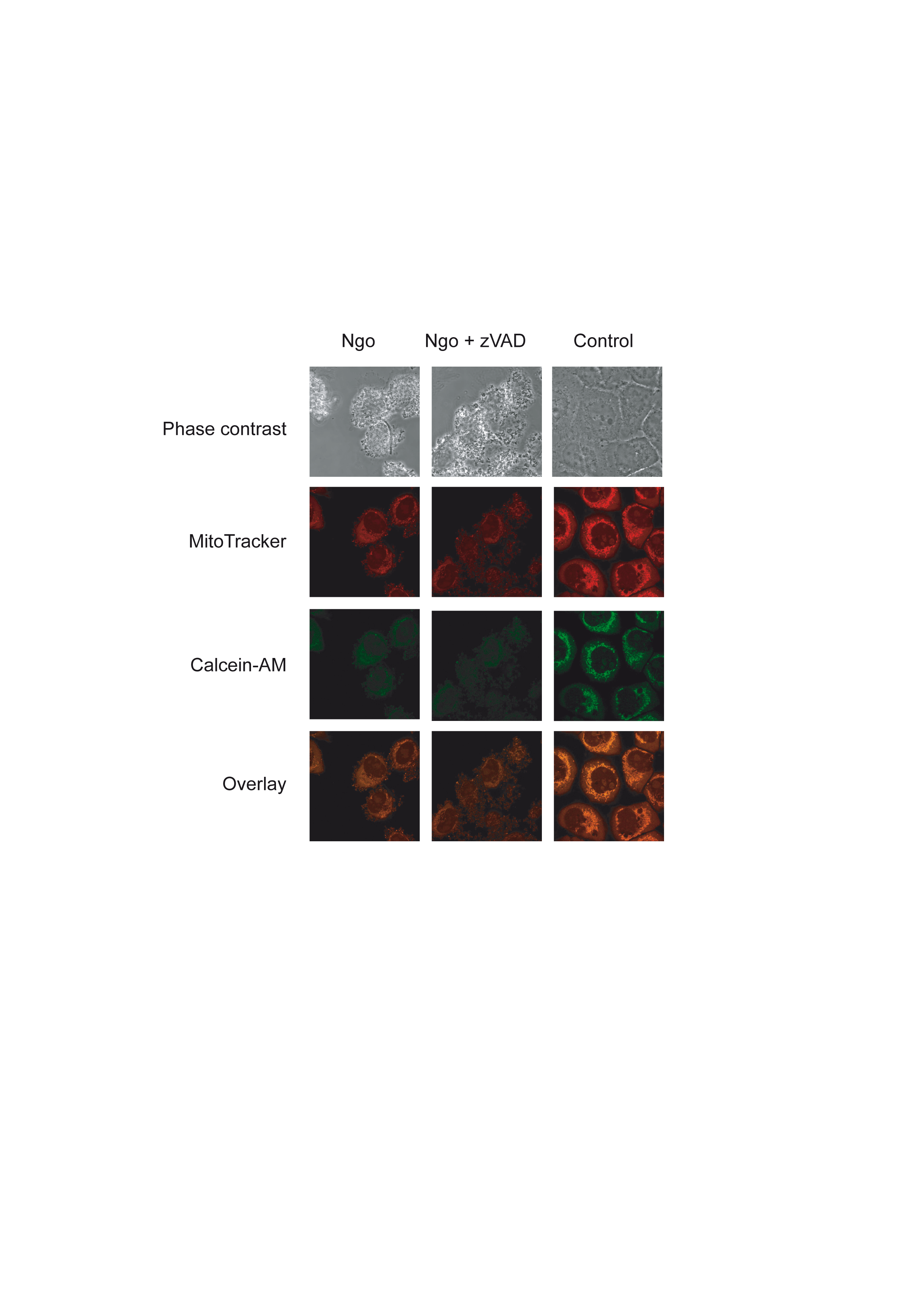

Supplement: Figure S5 — HeLa cells infected with strain N920 in the absence or presence of caspase inhibitor zVAD were loaded with calcein-AM (green) and CoCl2 as mentioned in Protocol S1. Cells were then incubated with MitoTracker Orange (red) to check for membrane potential loss. The respective overlays are shown. (2.39 MB TIF) [file ppat.1000629.s006.tif]

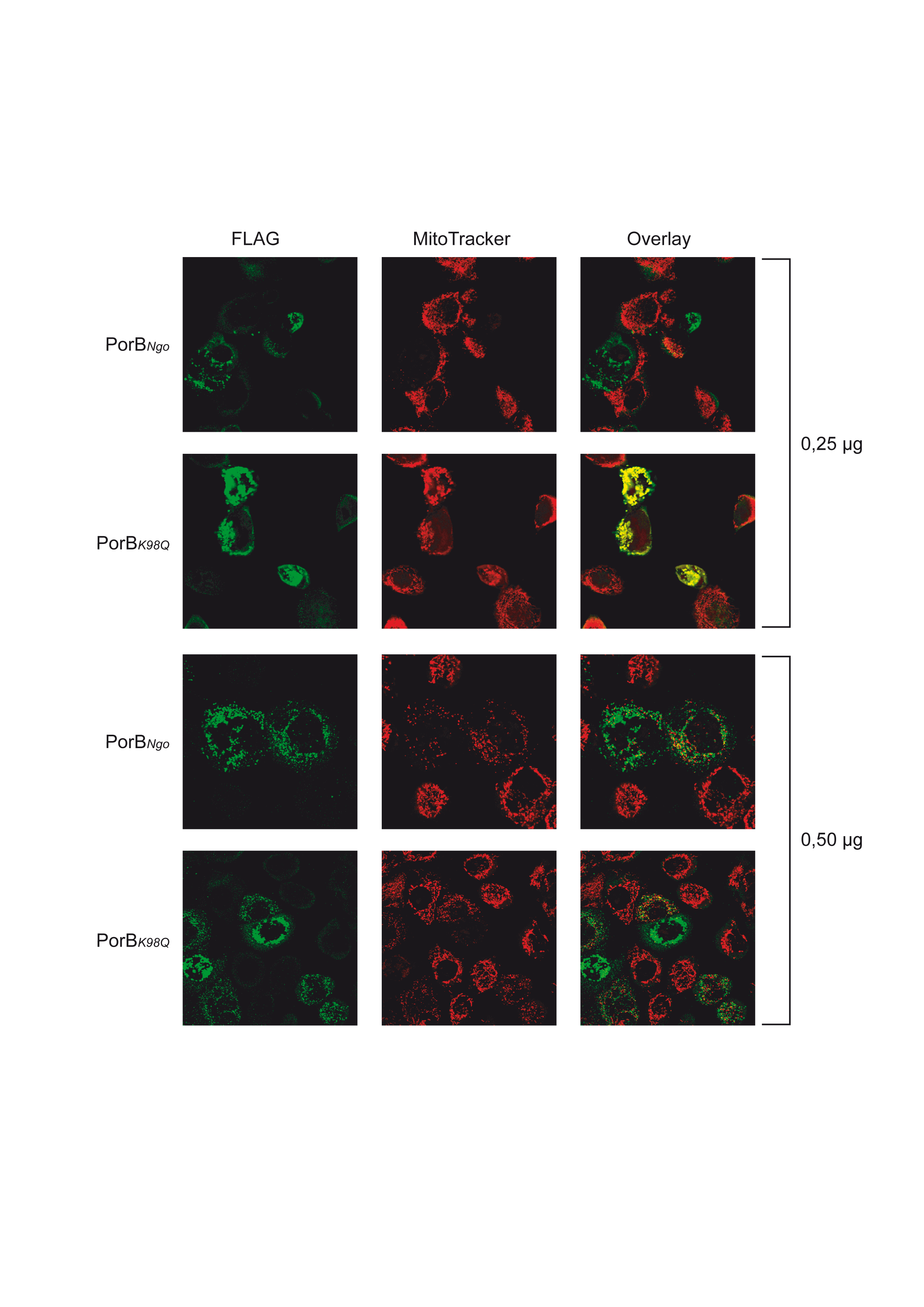

Supplement: Figure S6 — PorBK98Q is targeted to mitochondria upon overexpression. HeLa cells were transfected with expression constructs for wildtype PorB (PorBNgo) and ATP-binding mutant (PorBK98Q) at two different concentrations (0.25 and 0.5 µg/ml) and the expression of PorB (FLAG) and the presence of ΔΨm (MitoTracker) were monitored. (2.56 MB TIF) [file ppat.1000629.s007.tif]

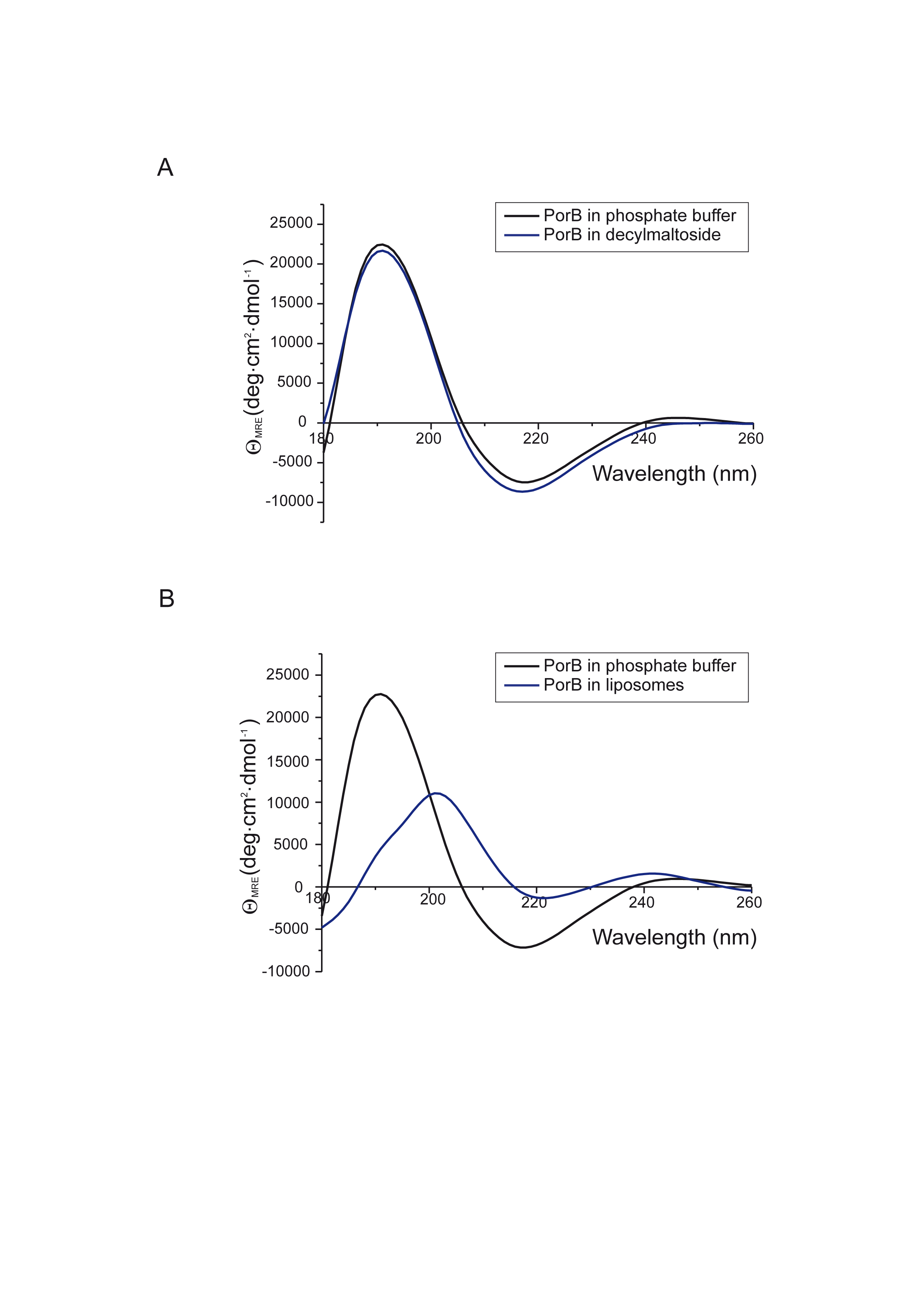

Supplement: Figure S7 — (A) An assessment of the secondary structure of PorB. CD spectra of PorB in solution (20 mM K2HPO4/KH2PO4) and of renatured PorB in decylmaltoside. (B) CD spectra of PorB in solution (20 mM K2HPO4/KH2PO4) and of renatured PorB in liposomes. Experiments were performed as described [13]. (0.78 MB TIF) [file ppat.1000629.s008.tif]
